# Nonpathological inflammation drives the development of an avian flight adaptation

**DOI:** 10.1073/pnas.2219757120

**Published:** 2023-05-01

**Authors:** Dana J. Rashid, Joseph R. Sheheen, Tori Huey, Kevin Surya, Jackson B. Sanders, John R. Horner, Jovanka Voyich, Susan C. Chapman

**Affiliations:** ^a^Department of Microbiology and Cell Biology, Montana State University, Bozeman, MT 59717; ^b^Department of Biological Sciences, Clemson University, Clemson, SC 29634; ^c^Molecular Biosciences Program, Montana State University, Bozeman, MT 59717; ^d^Schmid College of Science and Technology, Chapman University, Orange, CA 92866

**Keywords:** intervertebral disc, nucleus pulposus, necroptosis, heterophil, corticosteroid

## Abstract

The development of modern birds provides a window into the biology of their dinosaur ancestors. We investigated avian postnatal development and found that sterile inflammation drives formation of the pygostyle, a compound structure resulting from bone fusion in the tail. Inflammation is generally induced by compromised tissue integrity, but here is involved in normal bone development. Transcriptome profiling and immuno/histochemistry reveal a robust inflammatory response that resembles bone fracture healing. The data suggest the involvement of necroptosis and multiple immune cell types, notably heterophils (the avian equivalent of neutrophils). Additionally, nucleus pulposus structures, heretofore unknown in birds, are involved in disc remodeling. Anti-inflammatory corticosteroid treatment inhibited vertebral fusion, substantiating the crucial role of inflammation in the ankylosis process. This study shows that inflammation can drive developmental skeletogenesis, in this case leading to the formation of a flight-adapted tail structure on the evolutionary path to modern avians.

## Significance

Here, we describe the mechanism for a prominent avian evolutionary adaptation. We find that vertebral fusion in the avian tail is driven by sterile inflammation and resembles bone fracture repair. This finding demonstrates a role for inflammation in developmental skeletogenesis. The immune system is involved in pathological ankylosis, but we find that it is also a crucial contributor to normal vertebral fusion. Evidence of necroptosis indicates that this type of cell death can occur in postnatal development. Also, we document nucleus pulposus structures in tail discs in birds. Lastly, corticosteroid treatment inhibits vertebral fusion, suppressing a Cretaceous avian flight adaptation while also demonstrating an unreported effect of corticosteroids on skeletal maturation.

**Fig. 1.**
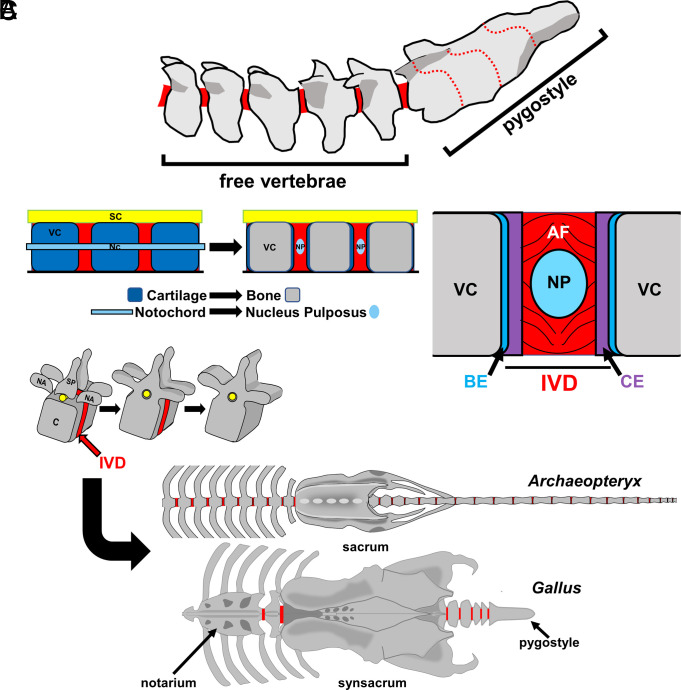
Tail morphology and vertebral fusion in extant and fossil birds. (*A*) *G. gallus* tail. Free, or unfused, caudal vertebrae are at the proximal end, and the distal caudal vertebrae are fused into the pygostyle structure. Solid red areas are IVDs, and dotted red lines indicate fusion planes within the pygostyle. (*B*) *Left*, nucleus pulposus (NP) formation diagram, sagittal view, from the embryonic notochord (NC) (*Left*) to postnatal NP (*Right*); SC: spinal cord. IVDs are red. *Right*, postnatal IVD diagram. The annulus fibrosus (AF) surrounds the NP, and these are encased in the cartilage end plates (CE) of the disc between vertebral centra (VC). Bone end plates (BE) are outside of the IVD. (*C*) *Top*, vertebral fusion diagram. Neural arch (NA) and spinous process (SP) elements fuse to form the neural arch, followed by ankylosis of the fused neural arch to the centrum (C). *Middle*, diagram of the *Archaeopteryx* spinal column (adapted from ref. 9); anterior to the *Left*. *Archaeopteryx* had 4 to 6 fused vertebrae in its sacrum. *Bottom*, diagram of the *G. gallus* spinal column, anterior to the *Left*. The chicken has 23 to 24 combined fused vertebrae in its axial column in the notarium, synsacrum, and pygostyle compound structures.

The avian spine has acquired multiple adaptations throughout bird evolutionary history. During the Mesozoic era, prominent adaptations included a shortened tail and distal vertebral fusion forming the pygostyle (1). The resulting morphology is a bipartite tail architecture with a proximal region composed of unfused, or “free” vertebrae, and a distal region comprising the compound pygostyle (Fig. 1*A*). There is evidence of convergent pygostyle evolution in avian and nonavian dinosaurs (2, 3), and the pygostyle was not always a flight adaptation. However, when allied with flight, the coevolution of the pygostyle and tail feather fan with a shortened tail (4–6) contributed to improved flight aerodynamics and control, with reduced drag, a more forward-distributed center of mass, and separation of the tail from the hindlimbs as its own locomotory unit (7, 8). Since avian pygostyle vertebral fusion has remained largely unchanged, we reasoned that the underlying mechanisms responsible for its emergence could be explored by analyzing tail development in modern birds.

The bird tail, like other axial regions in amniotes, forms as a string of vertebrae separated by intervertebral discs (IVDs) (10). The discs, in turn, are composed of several discrete tissues. The embryonic precursors of the vertebrae and the IVD cartilages are the somites. In mammalian embryos, the rod-like notochord fragments and its remnants form the nucleus pulposus (NP) structures in the centers of the discs (Fig. 1*B*). The fully formed mammalian IVD is composed of the cartilage end plates, the annulus fibrosus (AF), and the NP; together these function to provide spinal cushioning and flexibility. Previous research suggested that birds do not form NP structures (10, 11), a notion challenged by this study.

Vertebral development continues near birth/hatching with a series of bone fusion events, including fusion of the neural arch components and fusion of the neural arches to the centra to form individual vertebrae (Fig. 1*C*). In most terrestrial vertebrates, the neural arches and centra of neighboring vertebrae fuse together to form the sacrum. Because neighboring vertebral fusion occurs postnatally, after the IVDs and bony vertebrae have already formed, the ankylosis process requires the dismantling and remodeling of the discs to bone.

Vertebral fusion is more extensive in birds than in other vertebrates. From the Cretaceous period onward, thoracic, lumbar, and caudal vertebrae fuse into the sacrum to form the synsacrum, thoracic vertebrae fuse into the notarium (in some species), cervical vertebrae fuse to become syncervicals (as in hornbills), and the distalmost caudal vertebrae fuse forming the pygostyle (Fig. 1*C*). Like sacral fusion in mammals, pygostyle fusion occurs during juvenile development (12). Few mechanistic studies of normal vertebral fusion have been conducted, but for pygostyle fusion, our data show involvement of the immune system. These analyses reveal the ancestral amniote vertebral joint development in the avian tail, and a role for inflammation in developmental skeletogenesis.

## Results

To investigate vertebral fusion in the avian pygostyle, we first analyzed avian IVD anatomy and development in the tail. By histology, we found equivalent avian development to mammals, including notochord-derived NP structures. Evidence of immune response prompted whole transcriptome RNA sequencing (RNAseq) and reverse transcription polymerase chain reaction (RT-PCR) microarray analyses. Inflammation involving necroptosis and multiple immune cell types was strongly implicated, leading us to substantiate necroptosis and heterophil function at the protein level. Corticosteroid treatment was then performed to functionally test the involvement of inflammation in pygostyle fusion.

### Histochemical Analyses Implicate Immune System Involvement.

We evaluated avian IVD development and architecture relative to mammalian and investigated differences between fusing and nonfusing caudal discs. Chicken tails from embryos [E15 (embryonic day 15), E18, and E19 (hatching occurs at E20/21)] and posthatch chick tails [D8 (posthatching day 8) and collected every 2 wk thereafter until D105] were histologically stained and analyzed by standard or plane polarized light microscopy.

**Fig. 2.**
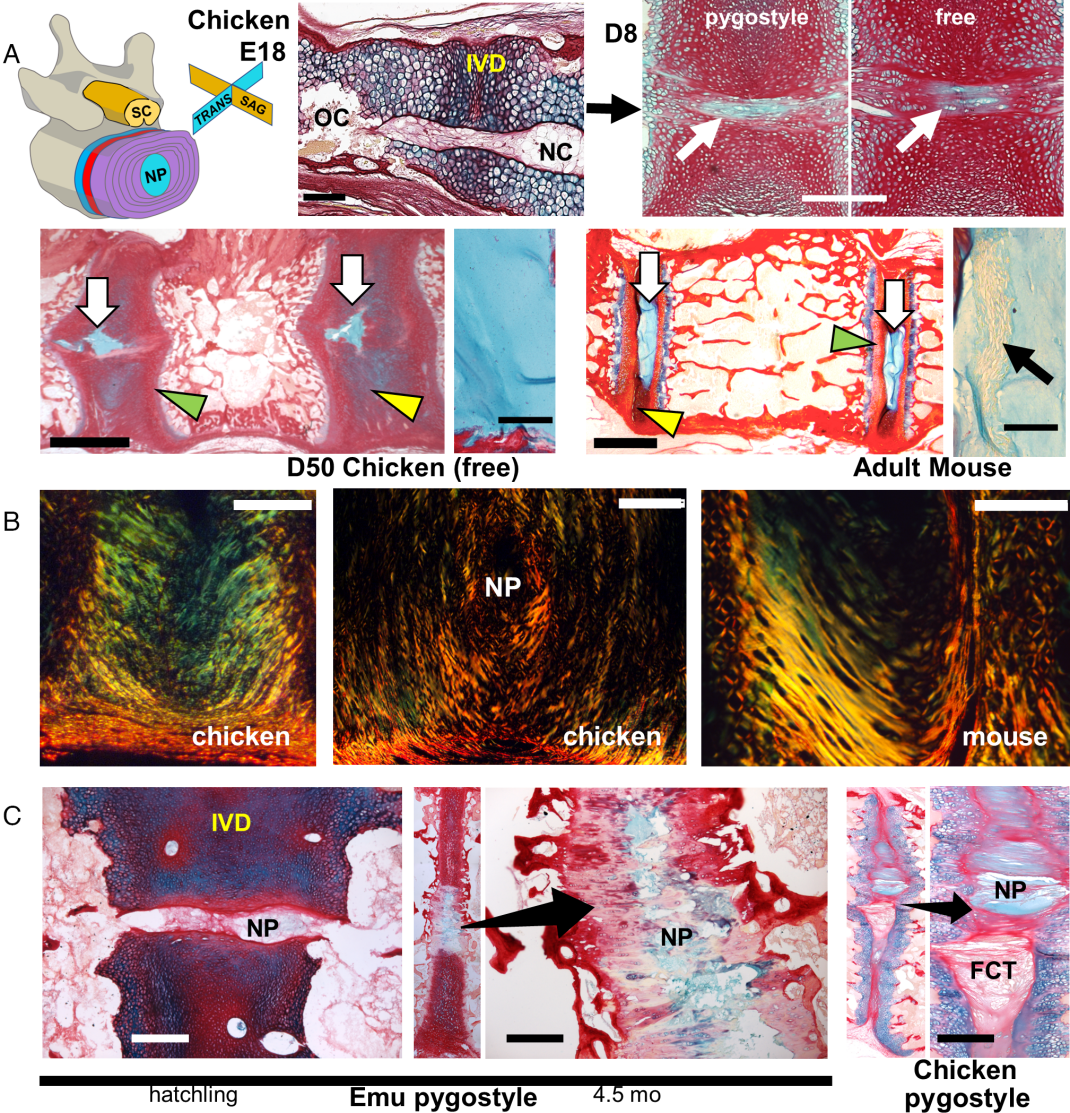
NP formation and IVD architecture in the avian tail. All panels are sagittal views, as noted in the schematic in (*A*), except for *(B)*, *middle* panel, transverse view. (*A*) NP development in chicken from E19 to D50 (ABPR staining). Notochord (NC) partitioning occurs at E19; OC are ossification centers in the centra; scale bar, 250 µm. At D8, cellular NPs are evident in both pygostyle discs (*Left*) and free vertebrae caudal discs (*Right*). Arrows indicate NPs; scale bar, 200 µm. At D50, NPs are acellular (scale bar, 2 mm) (magnified view of the NP to the *Right* of the panel, scale bar, 100 µm) compared to the cellular adult mouse NP (scale bar, 1 mm), right magnified panel, scale bar, 100 µm. NPs (white arrows), AF (yellow arrowhead), and CE (green arrowhead) structures. (*B*) Lamellar architecture of chicken and mouse IVDs by plane polarized light. *Left*, D50 chicken free tail, scale bar, 500 µm. *Middle*, D50 chicken, scale bar, 300 µm. *Right*, adult mouse, scale bar, 100 µm. (*C*) Pygostyle IVDs in the emu and chicken, ABPRH staining. In the emu hatchling pygostyle, IVDs with nascent NPs are observed (cryosection; scale bar, 250 µm). At 4.5 mo, proteoglycan-rich NPs in the pygostyle are evident in the full pygostyle IVD and in magnified view; scale bar, 250 µm. In chicken pygostyle IVDs, NPs and fibrous connective tissue (FCT) are evident; D50; scale bar, 250 µm.

Similar to NP formation in mammals, we found that the notochord in avian tails fragments and its remnants become the central disc NP (Fig. 2*A*). The previous studies that suggested that birds do not form NP structures did not evaluate tail IVDs (10, 11). In the chicken, notochord partitioning occurs near hatching, and fully formed cellular NPs are observed throughout the tail by D8. By D42, the NPs are proteoglycan rich but, unlike mammalian, are acellular. Even so, the free caudal discs greatly resemble mammalian discs, with cartilage end plates, NP, and lamellar annulus fibrosus structures (Fig. 2 *A* and *B*).

We found that NP formation in the tail occurs across avian clades, as evidenced by notochord partitioning in emu hatchlings and acellular NPs in emu juveniles (Fig. 2*C*). Emus are paleognathous birds, the group that is most genetically and morphologically distant from the other two modern bird groups, neoaves and galloanseriforms. Nuclei pulposi, therefore, are likely a universal avian trait.

A notable difference between pygostyle and free IVDs is pygostyle-specific intervertebral fibroconnective tissue (Fig. 2*C*). This tissue in pygostyle discs, observed by D21, is surrounded by fibrocartilage and juts into the IVD from the ventral side, terminating just below the NP. Its tissue fibers are contiguous with the fibrous layer of the periosteum, suggesting a periosteal origin. This connective tissue is essential because during the fusion process, it provides a route for vasculature and immune cells to enter the middle of the disc.

**Fig. 3.**
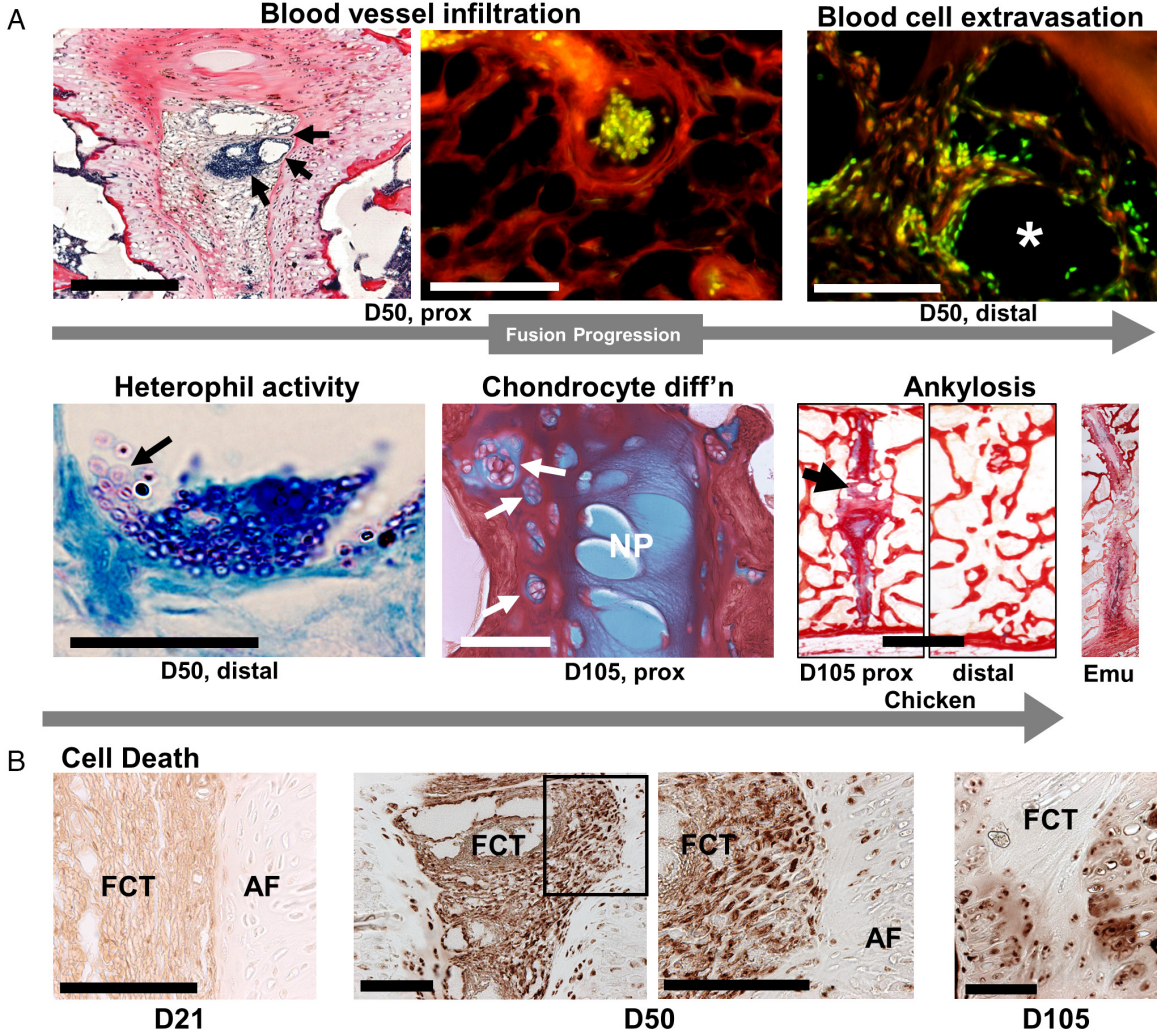
Pygostyle fusion events. All panels are sagittal views. (*A*) Progression of fusion. By D50, in the proximal disc, blood vessels (arrows) accumulate in the FCT below the NP; PRH staining; scale bar, 250 µm. In a magnified view, blood cells enclosed in a blood vessel are seen in the FCT before extravasation (eosin/SYTOX green staining; scale bar, 50 µm). Also, at D50 but in the distal disc, at a slightly later stage, blood cell extravasation into the FCT is observed (eosin/SYTOX green staining; the white asterisk indicates the center of a deteriorated blood vessel; scale bar, 50 µm). At D50, cells morphologically consistent with heterophils (8 to 10 µm cell diameter, bilobed nuclei) are observed (the arrow indicates a cluster of heterophils in the FCT; Giemsa–Wright stain; scale bar, 50 µm). By D105, chondrocyte differentiation to hypertrophic chondrocytes (arrows) is observed in the AF of the proximal pygostyle disc, ABPR staining; scale bar, 100 µm). Also, at D105 in the proximal disc, osteogenesis at the IVD center, where the NP had resided, is seen (noted by the arrow; scale bar, 2 mm). The distal disc has completely remodeled to bone. Similarly, ankylosis begins at the NP site in the emu; distal disc, 4.5 mo. (*B*) TUNEL assays; scale bar’s 100 µm. At D21, sparse cell death is detected (note background cytoplasmic FCT stain). By D50, in both discs, there is considerable cell death in the FCT and surrounding AF cells; TUNEL-stained nuclei are evident in the magnified boxed area. At D105, cell death is observed in the AF, below the NP, in hypertrophic chondrocytes.

Pygostyle formation entails remodeling of IVDs followed by bone formation in successive posthatching events that occur in the distal to proximal direction (12). As fusion progresses (Fig. 3*A*), the fibroconnective tissue expands and accumulates blood vessels ventral to the NP. Subsequently, blood-borne cells, including those with bilobed nuclei and consistent in size (8 to 10 µm) with heterophils (13), the avian equivalent of neutrophils (14), extravasate and migrate into the connective tissue. At D105, rafts of hypertrophic chondrocytes in the AF indicate fibrocartilage cell differentiation. At these later stages, ankylosis is most advanced in and around the NP, with bridges of cartilage tissue first traversing the NP region before complete disc remodeling to trabecular bone. Fusing discs in the juvenile emu pygostyle show a similar pattern, where the NP and surrounding AF are the first disc areas to undergo remodeling and osteogenesis events (Fig. 3*A*).

Using the TUNEL assay in chicken, we tested for cell death in disc remodeling [Fig. 3*B* and *SI Appendix*, Fig. S1*A*; (15–17)]. We found that cell death begins in the middle of the pygostyle discs, after D21, markedly in the fibroconnective tissue and surrounding AF, before expanding dorsally and ventrally in hypertrophic chondrocytes. Compared to D21 controls, we observed a 20-fold increase in TUNEL-positive cells at D50 (Fig. 5*B*). Cell death, therefore, was first observed at the same site and approximate timeframe as infiltration of blood vessels and extravasation of blood cells in the pygostyle discs. In one D50 specimen, scattered TUNEL-positive cells were observed in the absence of blood vessels (*SI Appendix*, Fig. S1*A*), suggesting that cell death may precede angiogenesis, though more investigation is required to substantiate this hypothesis.

These histochemical analyses show that 1) birds form NP structures in their caudal IVDs; 2) considerable cell death begins in the center of remodeling IVDs; and 3), there is accompanying infiltration of blood vessels and extravasation of blood cells, likely including heterophils, into the fibroconnective tissue. Together, these results suggested that pygostyle fusion involves immune response, possibly associated with cell death in the center of the disc.

### RNAseq and Corroborating Analyses Indicate Inflammation.

**Fig. 4.**
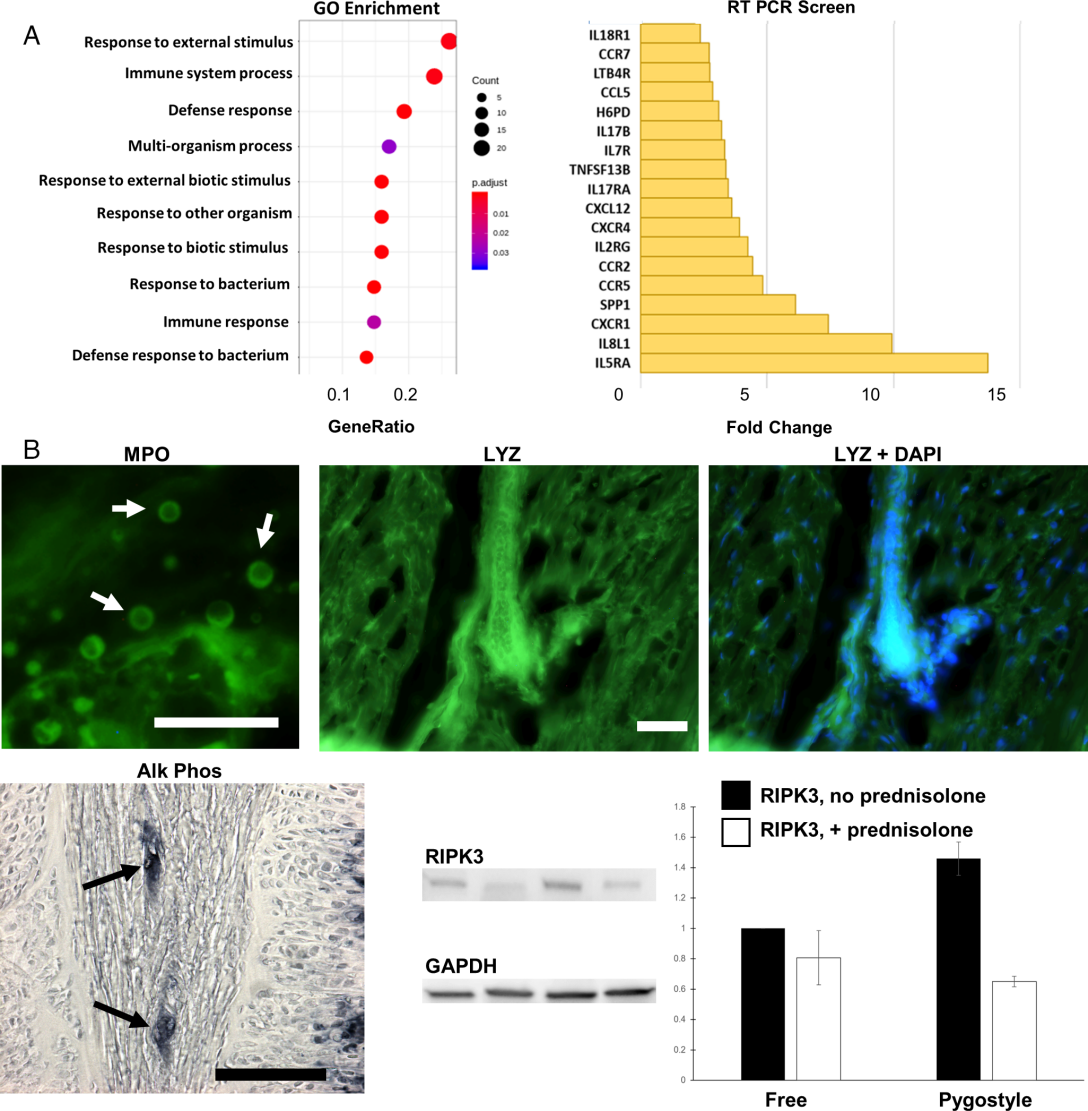
RNAseq data, supported by RT-PCR and protein analyses. (*A*) *Left*, all top 10 GO biological processes from the RNAseq profiling are immune-specific (*SI Appendix*, Table S3). *Right*, Profiler^2^ RT-PCR screen of chicken cytokines and chemokines, up-regulated genes. All RT-PCR differentially expressed genes were also differentially expressed in the RNAseq data. (*B*) Corroboration of the RNAseq data at the protein level. *Top*, IHC of MPO (myeloperoxidase; scale bar, 50 µm) and LYZ (lysozyme; scale bar, 50 µm) immunostaining of D50 chicken pygostyle IVD cryosections. DAPI nuclei stain with LYZ staining emphasizes detection of LYZ within blood vessels. *Bottom:* *Left*, AP activity stain of a D50 fusing pygostyle disc cryosection (scale bar, 200 µm); *Right*, anti-RIPK3 western blot and corresponding bar graph, showing that RIPK3 is more highly expressed in fusing pygostyle discs than in free discs, and its expression is inhibited upon prednisolone treatment.

To gauge the level of immune response and to identify other potential processes occurring during vertebral ankylosis, we performed whole transcriptome RNAseq profiling comparing remodeling pygostyle IVD tissue to free unfused caudal IVDs (four biological replicates for each condition). Illumina RNA sequencing was conducted, and the differentially expressed genes were analyzed by the Gene Ontology (GO) (18, 19) and the Kyoto Encyclopedia of Genes and Genomes (KEGG) (20) predictive algorithms and by manual gene-by-gene scrutiny. The screen yielded 2,087 significantly up-regulated and 1,412 significantly down-regulated genes in pygostyle fusion relative to control tissue (>twofold, *P* < 0.05) (see Dataset S1 for the specific fold changes; see also *SI Appendix*, Table S1 and Fig. S2 for data quality control parameters). GO and KEGG analyses indicate that amplification of immune response is the predominant differential activity occurring in remodeling pygostyle discs (Fig. 4*A* and *SI Appendix*, Tables S2–S4 and Figs. S2–S5). Upregulation of proinflammatory effectors and biomarkers involved in active inflammation was observed, including those in *IL-6*, *IL-1*, and *TNFα* pathways, and leukotriene synthesis and uptake (e.g., *LTC4SL*, *ALOX5AP*, and *SLC01B1*). *PTX3*, a C-reactive protein–related family member, was also up-regulated. Manual gene-by-gene scrutiny, utilizing https://www.genecards.org, revealed differentially up-regulated genes involved in heterophil/neutrophil (e.g., *CXCR1*, *MPO*, *CSF3R*, *LECT2*, *NCF1, 2*, and *4*) and monocyte/macrophage (e.g., *CAMP*, *CD163*, *CSF1R*, and *MBL2*) function. Transcripts predominantly expressed in T cells, dendritic cells, eosinophils, or platelets were also well represented. Evidence of phagocytosis, a primary function of neutrophils and macrophages, was observed with upregulation of *SYK*, *NCKAP1L*, *ITG2*, *S100A9*, and *RAC2*, among others (*SI Appendix*, Table S2). Notably, multiple members of complement and coagulation cascades were up-regulated, as well as osteogenesis-specific genes, such as *BMP* pathway members, *WNT* family members and their antagonists, and genes critical to osteoblast and osteoclast differentiation and function (*SI Appendix*, Table S6). Also noteworthy was upregulation of genes involved in necroptosis, including *RIPK3*, *MLKL*, and upstream toll-like receptors.

To affirm the integrity of our RNAseq results, we conducted RT-PCR arrays on fusing chicken pygostyle tissue using the Chicken RT^2^ Profiler system (Fig. 4*A* and *SI Appendix*, Table S5). Of the screen’s 96 cytokine and chemokine markers, 18 genes were significantly up-regulated and 11 significantly down-regulated (>twofold, *P* < 0.05) compared to free IVD controls. Up-regulated genes were enriched for heterophil, eosinophil, monocyte, and T cell function, osteogenesis, and overall enhanced immune response. All markers identified as either significantly up- or down-regulated in the RT-PCR arrays were also significantly differentially expressed in the RNAseq profiling, supporting the validity of the transcriptome data.

To confirm the RNAseq data at the protein level, and to substantiate key aspects of the fusion process, we tested for proteins associated with neutrophils and necroptosis (Fig. 4*B* and *SI Appendix*, Fig. S1). Myeloperoxidase (MPO), a marker for activated mammalian neutrophils, was observed in cells consistent in size with heterophils (8 to 10 µm diameter) in the fibroconnective tissue of fusing pygostyle IVDs, within or near blood vessels. Lysozyme (LYZ), a protein highly expressed in mammalian neutrophils and chicken heterophils (21, 22), was also observed in the fibroconnective tissue, in blood cells within blood vessels. MPO and LYZ are among the most differentially up-regulated genes in our RNAseq (11.0- and 16.4-fold, respectively). Neither were found in free IVDs, consistent with the lack of blood vessels in these nonfusing discs (*SI Appendix*, Fig. S1). Alkaline phosphatase (AP), up-regulated in our RNAseq, is an enzyme expressed in myeloid lineage blood cells (including neutrophils, monocytes, platelets, and other innate immune cells) (23, 24). AP activity was observed within blood vessels in the fibroconnective tissue by NBT/BCIP staining (Fig. 4*B*). To test for necroptosis-specific proteins, we analyzed RIPK3 expression, up-regulated 12.3 fold in the RNAseq, by western blot. RIPK3 protein was found to be differentially up-regulated 1.5-fold in pygostyle fusing discs compared to free discs (Fig. 4*B*). These protein-specific analyses support the RNAseq and microarray data, and together these implicate sterile inflammation involving necroptosis.

### Corticosteroid Treatment Inhibits Ankylosis.

**Fig. 5.**
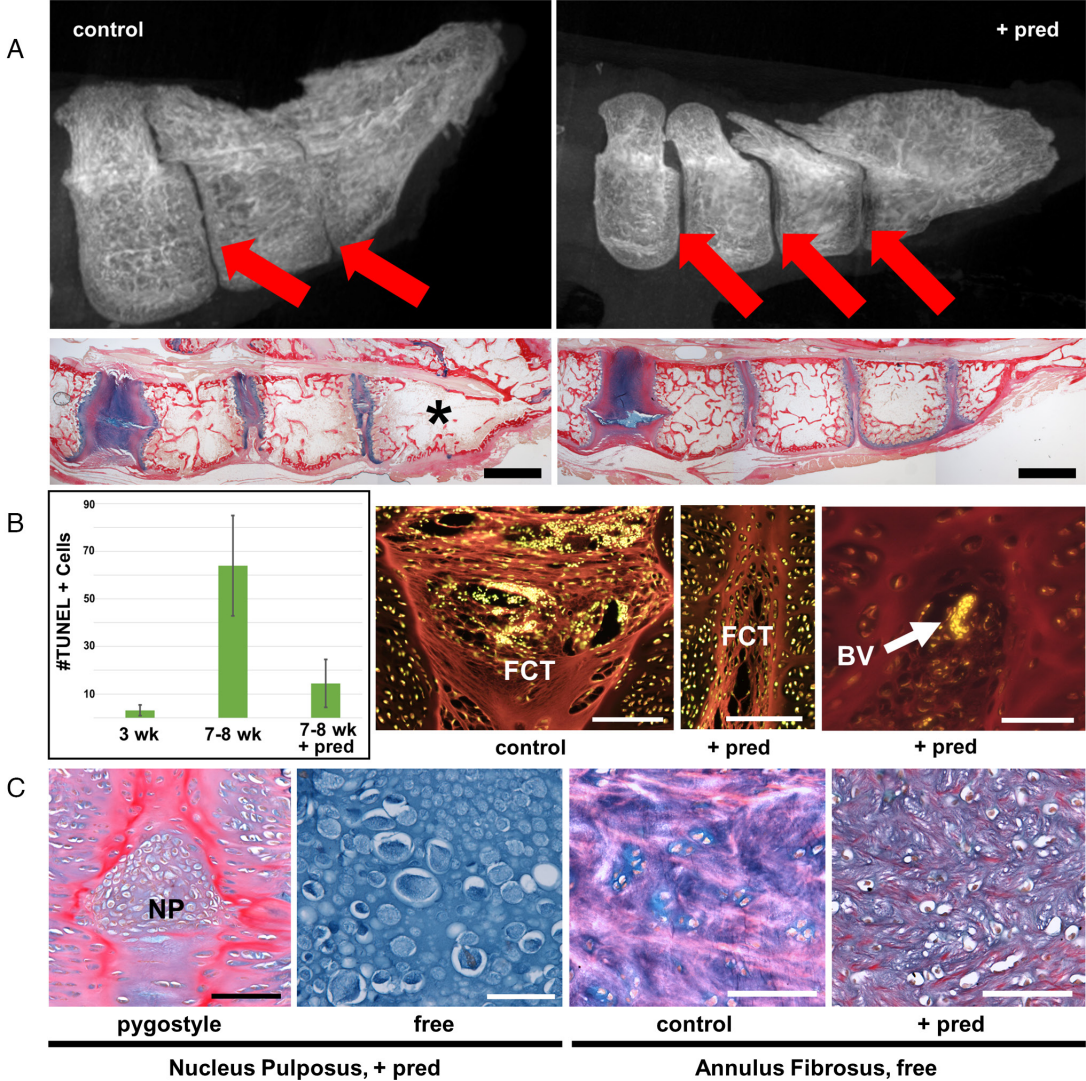
Prednisolone inhibits pygostyle fusion. Chicken, panels in sagittal view, distal to the *Right*. (*A*) Ankylosis inhibition. *Top*, microCT of 8-wk-old control (*Left*) and prednisolone-treated (*Right*) pygostyles. Red arrows indicate IVDs. Below, corresponding ABPRH-staining of the same respective conditions; the asterisk indicates the fused distalmost IVD region; scale bar’s, 2 mm. Note that pygostyle discs are thinner than free vertebrae discs. (*B*) Prednisolone inhibition of cell death and inflammatory events. *Left*, TUNEL analysis of (*Left* to *Right*) 3-wk-old controls, 7- to 8-wk controls, and 7- to 8-wk prednisolone-treated IVDs. In the next three panels (all at 8 wk of age), compared to untreated controls (*Left*, Scale bar, 100 µm), prednisolone treatment (for 5 wk) inhibits FCT expansion (*Middle*, Scale bar, 100 µm) and blood cell extravasation into the FCT (*Right*, Scale bar, 50 µm); eosin/SYTOX green staining; images show red-stained FCT below the NP and green/yellow-stained nuclei. (*C*) Prednisolone effects on NP viability and debris clearance. Prednisolone treatment preserves NP cells in both pygostyle and free discs in the 8-wk-old pygostyle (*Left* 2 panels; compare to the acellular chicken NP in Fig. 2*A*; scale bar’s 100 µm and 50 µm, respectively); ABPRH staining. Prednisolone treatment (7 wk of age/4 wk prednisolone) also causes accumulation of cell debris in free caudal IVDs in AF tissue compared to 7-wk-old controls (*Right* 2 panels); ABPRH staining; scale bar’s 100 µm.

We inhibited inflammation with prednisolone, a corticosteroid. Prednisolone was orally administered to chickens from 3 to 8 wk posthatch, spanning the timeframe of fusion of the distalmost pygostyle disc. Substantiating our hypothesis that inflammation drives pygostyle fusion, 57% (8 out of 14 total) of prednisolone-treated birds at the 8-wk timepoint failed to fuse their distalmost pygostyle IVD, compared to 100% fusion from 6 wk onward in the untreated controls (n = 2 at 6 wk, n = 2 at 7 wk, and n = 4 at 8 wk) (Fig. 5*A*; of note, of the dozens of pygostyles we have analyzed at D49 to D56 in past years, all had complete fusion of the two distalmost pygostyle vertebrae). This result demonstrates a previously undocumented phenomenon in which inflammation drives a normal musculoskeletal developmental event. Treated birds exhibited the normal timeframe for other developmental events, including feather maturation and sexual traits. No pygostyle fusion differences were observed between males and females.

Prednisolone treatment caused anti-inflammatory-type effects and influenced other events in tail maturation. Anti-inflammatory changes in the fibroconnective tissue included reduced tissue expansion, reduced accumulation of blood vessels below the NP, and inhibited extravasation of blood cells into the tissue (Fig. 5*B*). Cell death inhibition in the fibroconnective tissue (by 4.5-fold) was observed by TUNEL (Fig. 5*B*) and corroborated by inhibition of RIPK3 expression with prednisolone treatment (Fig. 4*B*). Together with the significant increase in *RIPK3* and *MLKL* expression in our RNAseq, these findings suggest that necroptosis is the predominant mechanism of cell death. Of note, in controls, the NP was acellular by 6 wk of age. However, in 8-wk prednisolone-treated birds, viable NP cells were observed throughout the entire tail (Fig. 5*B* and *SI Appendix*, Fig. S1). In addition, buildup of cellular detritus was evident in the AF of free caudal IVDs (Fig. 5*B*). Prednisolone, therefore, affected multiple normal maturational events in posthatch tail development.

## Discussion

Vertebral fusion of the bird tail necessitates that the pygostyle IVDs are remodeled to bone. Since multiple discrete disc structures are involved in this process, we followed the development and morphogenetic changes in these structures from hatching to the completion of fusion. Our surprising findings lend insight into avian evolution and postnatal spine maturation.

Early in our investigation, we found that chicken caudal IVDs and mammalian IVDs follow the same overall developmental and architectural plan, including NP formation. By following the fate of the notochord, we confirmed true notochord-derived NPs in the tail. NPs have been identified in numerous living and extinct amniote groups, including nonavian dinosaurs (10). Their presence in paleognathous and neognathous birds corroborates the concept that the ancestral amniote vertebral joint formed with a central NP (25). A likely scenario is that the avian tail has retained the ancestral disc development program, whereas the more anterior regions of the vertebral column have lost the NP as a result of subsequent adaptations.

Several inflammatory-like phenomena observed in the histological survey, particularly the presence of heterophils, implied that inflammatory response was involved in pygostyle fusion. Classically, inflammation is the immune response to disease or trauma, often initiated by cell death, involving angiogenesis, recruitment and extravasation of immune cells to the target site, and upregulation of proinflammatory effectors. Analogously, here we observe substantial cell death in the intervertebral fibroconnective tissue and AF, coevident with angiogenesis and extravasation of immune cells, including heterophils, into the connective tissue, all consistent with inflammation. Also consistent with inflammation is the extensive upregulation of proinflammatory genes as indicated in our RNAseq profiling. Results indicate strong genetic signatures for activated phagocytosing neutrophils (heterophils in this case) and monocytes/macrophages, as well as upregulated genes involved in T lymphocyte, eosinophil, dendritic cell, and platelet function, providing additional evidence of a robust inflammatory response.

Pygostyle fusion, however, is a developmental process, and inflammation is rare in development. There is also no evidence of neutrophils or inflammatory response in normal endochondral ossification (26) (*SI Appendix*, Fig. S6*A*). Developmental roles for macrophages and macrophage-related cells have been identified, including synaptic pruning, interdigital tissue removal, and the remodeling of cartilage models to bone (27, 28), though these processes generate a neutral or anti-inflammatory transcriptional signature (29).

More comparable developmental processes are formation of the external egg anchoring plug in ricefishes (30) and mammalian embryo implantation (31), both with proinflammatory cascades and evidence of neutrophil involvement. A significant commonality shared among these processes and pygostyle fusion is vascular remodeling lured by tissue damage or death. Blood vessels infiltrate the forming ricefish egg plug upon egg fiber-mediated tissue damage (30); uterine-trophoblast contact-mediated tissue damage initiates a uterine capillary network around the implanting mammalian embryo (31, 32); and blood vessels elaborate beneath the NP in pygostyle fusion, associated with fibroconnective tissue cell death. A major function of inflammation in vertebrates is to bring blood vessels and immune cells to sites of pathology, and for these three postnatal processes, their need for angiogenesis is met by inflammation. Our collective data, providing multiple lines of evidence that show sterile inflammation underlies pygostyle fusion, expand the known roles of inflammation in development.

MPO and LYZ staining in fusing disc tissue further substantiate heterophil phagocytic activity. In mammals and zebrafish, MPO and LYZ are concentrated in neutrophil granules, which are released upon targeting of neutrophils to the site of inflammation (33). In chicken heterophils, LYZ is highly expressed but MPO expression is controversial (34, 35), possibly due to sampling differences. We examined active heterophils in tissue, but most studies involve blood-isolated circulating heterophils. One theory posits that avian heterophils lack MPO and thus only minimally rely on oxidative mechanisms during inflammatory response (36). Our RNAseq data, however, show upregulation not just of *MPO* but also *CYBA*, *CYBB*, *NCF2*, *NCF4*, and *RAC2* genes specific for neutrophil oxidative burst. Our study and others (37, 38) indicate that similar to mammalian neutrophils, the release of reactive oxygen species is a significant factor of avian heterophil function.

*RIPK3* and *MLKL* upregulation indicates necroptosis, which likely accounts for the cell death we observed in fibroconnective tissue and AF cartilage. Toll-like receptors *TLR3* and *TLR4*, upstream inducers of necroptosis in mammals (39), were also up-regulated. RIPK3 and MLKL form the necrosome complex that increases plasma membrane permeability in affected cells, ultimately leading to cell death. Necroptosis, unlike apoptosis, can elicit an inflammatory response (40). Accordingly, the caspase-specific cascade typical of classic apoptosis is not observed in our RNAseq data. Necroptosis is involved in multiple inflammatory pathologies and is actively suppressed during embryonic development (39). Evidence of necroptosis in pygostyle fusion indicates that this form of cell death can operate in postnatal development.

Our corticosteroid functional analysis provides additional confirmation of the driving force of inflammation in disc remodeling and ankylosis. Corticosteroids are potent anti-inflammation drugs that suppress the immune system by sequestering and inhibiting T lymphocytes, inhibiting cytokine and lymphokine production, and inhibiting leukocyte migration (41), including extravasation of neutrophils into inflamed tissue (42). In bone, corticosteroids inhibit osteogenesis and delay bone fracture healing (43, 44), due to their impacts on the immune system and the balance of osteoblast and osteoclast-derived bone homeostasis (45). In this study, prednisolone’s anti-inflammatory effects were evidenced by inhibition of cell death, connective tissue expansion, infiltration of blood vessels into that tissue, and subsequent extravasation of blood cells. Inhibiting ankylosis was likely a downstream consequence of inflammation suppression and the effects of prednisolone on osteoblast and osteoclast function. Prednisolone treatment also impeded the clearance of AF cellular debris and resulted in retention of viable NP cells throughout the tail. The mechanisms responsible for these latter phenomena are unclear, but AF detritus buildup is likely due to inhibition of phagocytosis. Interestingly, in mice engineered without macrophages, capillaries in the retina that would normally undergo apoptosis instead are retained (reviewed in ref. 27), providing another example of tissue preservation upon immune system inhibition. In another relevant study, mice engineered to overexpress Sonic Hedgehog (SHH) in the NP failed to fuse their sacral vertebrae, suggesting a link between the NP and vertebral fusion (46). Investigation of the effects of corticosteroids on vertebral fusion in other axial regions is a focus of our ongoing studies.

We questioned whether any parallels could be drawn between pygostyle fusion and pathological instances of inflammation-driven osteogenesis, such as the disease ankylosing spondylitis and bone fracture repair. Comparisons with the PGISp ankylosing spondylitis mouse model (47), for example, show noteworthy overlap with our RNAseq data (*SI Appendix*, Table S6). In particular, neutrophil-enriched genes, complement, toll-like receptor signaling, and equivalent cytokines/chemokines are shared, although *WNT* signaling was distinctly different.

With bone fracture healing, we observe more numerous similarities. Direct comparisons of our RNAseq data and bone fracture healing mouse transcriptomes (48) show considerable overlap of inflammatory and osteogenesis genes (*SI Appendix*, Fig. S6). Diagnostic biomarkers of fracture repair are observed in pygostyle fusion, including *CD55*, *TLR4*, *IL6R*, *IL8*, and *C5AR1* (49). Complement and coagulation cascades are shared features, as are upregulation of metalloproteinases, changes in collagen isotypes (50), differentiation and subsequent cell death of hypertrophic chondrocytes, and inhibition by prednisolone (51). Periosteal-derived fibroconnective tissue involvement is another prominent phenomenon in both processes (52). In bone fracture repair, the periosteum, composed of fibroconnective and cambium layers, expands to form the callus, a connecting tissue bridge at the site of the break. Similar to pygostyle fusion, the callus is a conduit for blood vessels and immune cells and is a primary site of inflammation (52). Evidence of bone fracture repair traces back to early vertebrates in the Carboniferous (53); it follows that this ancient process could serve as the foundation for a subsequent evolutionary adaptation.

Several questions remain as to the precise initiating sequence of events in pygostyle fusion. We first observe fibroconnective tissue in soon-to-be fusing discs; whether this is a direct or indirect cause of fusion is unclear. Why the fibroconnective and AF tissues die is unknown, but inhibition of cell death by prednisolone suggests that the death is tied to the immune system. Considering the proximity of the NP to the fibroconnective tissue changes, the bone remodeling events occurring at the site of the NP, and the SHH link already established with vertebral fusion (46), the NP may serve as a signaling center for the fusion process, but this remains to be tested. Once necroptosis has occurred, the subsequent events mirror bone fracture repair. The numerous parallels between bone fracture healing and pygostyle formation lead us to conclude that a bone repair-type mechanism was co-opted during avian evolution to generate the pygostyle.

In summary, chicken pygostyle fusion is driven by sterile inflammation and involves nuclei pulposi and necroptosis. These findings are relevant to the evolution of birds and to the roles of the immune system in development. During avian evolution, the Pygostylian birds emerged in the Cretaceous with a shortened tail capped with a distal pygostyle. The inflammatory mechanism that drives formation of the modern bird pygostyle suggests that in the Mesozoic era, an evolutionary change that capitalized on a bone fracture repair strategy led to this important and enduring avian flight adaptation. The discovery of NP structures in avian tails has further implications for the evolution of the avian body plan, suggesting that the tail retains the plesiomorphic developmental program, and subsequent departures from that program occurred in more anterior axial regions. In the broader context of immune system roles in development, these findings indicate that inflammation can drive developmental skeletogenesis. Additionally, necroptosis functions in postnatal development, and corticosteroid treatment can inhibit vertebral fusion. These studies expand the known interactions between bone and the immune system, attesting to evolution’s wide-ranging mechanistic capacity for morphological change.

## Materials and Methods

### Key Resources.

For a source list of biological samples, reagents, software programs, deposited data, and reference databases, see *SI Appendix*, Table S7.

### Animal Models and Biological Samples.

#### *Gallus gallus*.

White Leghorn fertilized eggs (Charles River) were incubated to the appropriate developmental stage at Montana State University. D50 Cornish Rock carcasses were obtained from a local farm. Fertilized Bovan Brown and Tetra Brown chicken eggs were sourced from the Clemson Poultry Farm. Tetra maturation is delayed relative to the other breeds, so these birds were harvested at 10 wk instead of 8 wk; their maturation level was histologically assessed by the degree of pygostyle fusion. All breeds exhibited the same overall pygostyle fusion events, indicating that the process is consistent across subspecies. Eggs were incubated at 38 °C with automatic egg turning until the desired level of development and, if processed before hatching, were harvested into PBS and fixed in 4% paraformaldehyde.

#### *Dromaius novaehollandiae*.

Frozen emu hatchlings and a 4.5-mo-old carcass were obtained from the Montana Emu Ranch in Kalispell, MT.

#### *Mus musculus*.

Adult mouse carcasses (Balb/c) were obtained from the MSU Animal Resources Center. Thoracic spinal tissue was dissected and processed for histology.

All animal-related procedures complied with animal use protocols at Montana State University (approved IACUC protocol #2018-82) and at Clemson University (IACUC protocol #2019-047).

### Method Details.

#### Histology.

Chicken tail tissue was collected at E15, E18, and E19 (White Leghorn), at D8 (Bovan Brown), and then every 2 wk after hatching until D105 (Bovan Brown); the D50 timepoint was collected from Cornish Rock carcasses. Emu tail tissue was extracted from hatchling and 4.5-mo-old frozen carcasses and fixed with 4% paraformaldehyde. E18 and older tissue was subsequently demineralized by EDTA (54) and then either paraffin-embedded and sectioned (12) or cryosectioned as previously described (55). Alcian blue and picrosirius red (ABPR) staining on paraffin-embedded tissue was performed as in ref. 12. When hematoxylin was added to ABPR staining (ABPRH), Gill’s hematoxylin staining was performed after the acidified water step. Picrosirius red and hematoxylin (PRH) staining was conducted using the same protocol but omitting the alcian blue step. Wright–Giemsa staining on dewaxed paraffin sections was performed for 1.5 h at 37 °C, followed by washing, EtOH and xylenes dehydration, and then mounting in DPX. For eosin and SYTOX green staining on dewaxed paraffin sections, eosin (0.25% eosin Y in 80% EtOH/0.5% glacial acetic acid) was followed by SYTOX green staining (5 mM in DMSO stock; 0.2 µL SYTOX green stock in 1 mL PBS; 30 min at room temperature) before washing, dehydration, and mounting in DPX. For plane polarized light imaging, ABPR-stained sections were imaged using a NIKON Optiphot2-POL Polarizing Microscope, a Nikon DS-Fi2 Camera, and Nikon NIS-Elements Basic Research Image Capture analysis software. All other imaging of paraffin and cryosections was performed on a Zeiss Axioscope A.1 microscope in conjunction with a Jenoptik ProgRes C14 Plus digital camera and accompanying software.

#### TUNEL assays.

TUNEL assays were conducted on dewaxed paraffin slides of 3-wk-old (Bovan Brown), D50 (Cornish Rock, Bovan Brown), and D105 (Bovan Brown) chicken tail tissue using the In Situ Cell Death POD kit, including omitted TdT enzyme negative controls and DNAse-treated positive controls (*SI Appendix*, Fig. S1). Endogenous peroxidases were blocked with 0.3% H_2_O_2_ (room temperature, 30 min), permeabilized (0.1% Na citrate/0.1% TX-100) and blocked in NGS blocking buffer (0.03M Tris pH 7.5, 0.15M NaCl, 1% glycine, 0.4% Triton X-100, and 10% goat serum) to limit background connective tissue cytosolic staining. Labeling was performed according to the manufacturer’s instructions, followed by DAB staining, washing, and mounting.

#### Illumina mRNA sequencing.

Total RNA from dissected pygostyle and free IVDs from eight 10-wk-old Tetra breed chickens (equivalent to D50 of other breeds used) was isolated by TRIzol/chloroform phase separation and quantified using the Qubit RNA HS kit. The mRNA was enriched by polyA capture using the NEBNext® Poly(A) mRNA Magnetic Isolation Module followed by library preparation using the NEBNext® Ultra™ II RNA Library Prep Kit for Illumina®. The library was unstranded. The sequencing was performed on a NextSeq 500/550 Mid Output Kit v2.5 (300 Cycles) with prepped libraries from eight samples (four free caudal IVDs and four fusing pygostyle IVDs). The NextSeq 550 uses RTA2 (Real-Time Analysis 2) software to filter and assign quality scores to each base call. Default RTA2 filtering parameters were used, and only reads that passed filtering were used in downstream analysis. All fastq files were screened using FastQC for per base sequence quality, and for all reads, mean and median quality scored in the “very good” range (28 to 36) with minimum falloff near the ends of reads (the last 5 to 10 bases). The falloff reads scored in the acceptable “intermediate” quality range (20 to 28). The bioinformatic analysis was performed on a Linux platform utilizing a custom bioinformatics pipeline that included STAR (version 2.70f) alignment of reads, the SUBREAD featureCounts program (version 2.0.0) to produce count tables, and DESeq2 R software package (1.26.0; R version 2.6.3) for differential expression analysis. The analysis was conducted gene-wise. Mappings for multiple splice variants were combined; however, the featureCounts program excludes reads multimapping to different genes or duplicate exons. Default STAR mapping parameters for paired-end samples were used. The index was created from RefSeq GCF_000002315.6_GRCg6a_genomic.gtf and .fna files. The adapters were not removed, and reads were not trimmed for quality as STAR software uses local alignment and auto/soft trims reads during alignment.

See the *SI Appendix* file for comparisons of our RNAseq data to the PGISp mouse (47) and to mouse bone fracture healing transcriptomes (48).

#### RT-PCR array analysis.

Chicken pygostyle disc tissue was screened for chicken cytokines and chemokines using the Qiagen RT^2^ Profiler Chicken Chemokine PCR Array. Fusing pygostyle disc tissue and free caudal disc tissue from Bovan Brown chickens at 8 wk were harvested by dissection, and total RNA was isolated by TRIzol/chloroform phase separation and quantified using the Qubit RNA HS kit. RNA was isolated from four different batches of chicken tissue (from four chickens per batch), using free caudal IVD tissue for controls. Qiagen qPCR arrays were then performed according to the manufacturer’s instructions, and data analysis followed Qiagen guidelines.

#### Protein expression analysis.

Protein expression was investigated by immunohistochemistry staining (IHC), enzyme activity, and western blotting. IHC was performed on Dent’s fixed (80% MeOH, 20% DMSO) demineralized D50 chicken tail tissue cryosections. The sections were treated with 6% H_2_O_2_ (60), blocked, and antibody-stained with either the MPO or LYZ antibody (1:100 dilution); LYZ staining was followed by Alexafluor 488-conjugated secondary antibody staining. AP enzyme activity was detected on cryosections of demineralized D50 pygostyle disc tissue by NBT/BCIP staining (~2 min) followed by washing and mounting. For RIPK3 western blotting, 7.5-wk fusing pygostyle and nonfusing free chicken disc tissue (from four prednisolone-treated and four control birds, two males and two females in each group) was collected, homogenized, and normalized to 20 µg/µL. The blots were incubated with either the anti-RIPK3 antibody (1:1,000) or the anti-GDPH antibody (1:5,000; as a loading control), followed by secondary antibody incubation and ECL detection.

#### Prednisolone treatment.

Straight-run commercial broilers were obtained by the Clemson Poultry Farm and raised using a standard 14/10 light/dark cycle. Birds were split into three groups: males (15), females (9), and controls (14 mixed), kept in group pens and fed ad libitum with unrestricted access to water. From 3 to 8 wk of age, birds were dosed daily with orally administered prednisolone (1 mg/kg). Dissected tail tissues from prednisolone-treated and control birds were assessed for distalmost pygostyle IVD fusion by microCT analysis and histology, and in some cases, the IVDs were harvested for Western analysis. Distalmost IVDs were judged as unfused if more than half of the disc was evident; all but two of the tails were either fully fused or fully unfused.

#### microCT analysis.

Formalin-fixed pygostyles from control and prednisolone-treated broilers were analyzed by microcomputed tomography using a Bruker SkyScan 1176 (Godley-Snell Research Center, Clemson, SC, USA) at 50 kV, 486 µA current with a 0.2-mm Al filter, at 35-nm resolution. Images captured using the SkyScan 1176 were applied to NRecon reconstruction and CTVox volume-rendering software programs.

### Quantification and Statistical Analysis.

#### Histology and TUNEL analyses.

Three chicken tails each were histology-analyzed for E18, E19, D8, and D105 stages, and 10 tails were analyzed at D50. For TUNEL stains (n = 3 animals per timepoint, including controls), TUNEL-positive cells were counted using a grid (1360 × 1024 pixels) spanning the IVD region, at the same magnification (n ≥ 18 grids per condition). TUNEL data were plotted (using Microsoft Excel) by the average number of TUNEL-positive cells/panel, including SD values.

#### Sequencing and differential expression analysis.

RNAseq was performed using four biological replicates of each condition (fusing and free IVDs) on an Illumina NextSeq 550 mid output flow cell. Sequencing and basic Illumina filtering for quality reads produced a total of 172.08 million mappable reads averaging 302 base pairs per read across all samples. Statistical analysis for differentially expressed genes was performed using the recommended DESeq2 parameters, and only genes with a BH adjusted *P*-value (*P*adj) under 0.05 were considered significant. A correlational heatmap was generated to assess variation across all samples (*SI Appendix*, Fig. S2).

#### GO enrichment and KEGG pathways.

The David functional annotation tool version 6.8 was used to group all genes up-regulated over 2.0-fold in the RNA sequencing experiment with *P*-values less than 0.05 into both KEGG pathways and GO biological processes (*SI Appendix*, Tables S2 and S3). The input Gene list included 2,086 genes; those applied to the GO biological processes and KEGG pathways were included in the tables only if the BH adjusted *P*-value was below 0.01. A heatmap with GO enrichment for a restricted background reference library was also conducted (*SI Appendix*, Fig. S3 and Table S4).

#### RT-PCR.

Four biological replicates each were analyzed for fusing pygostyle and nonfusing free IVD conditions. All samples passed quality control using the RT^2^ Profiler RNA QC PCR array (PAHS-999Z). Fold-change was calculated as the normalized gene expression in the pygostyle samples divided by the normalized gene expression in the free disc control samples via delta delta Ct, using two genes selected from the full cytokine panel with the least change in Ct across all samples as housekeeping genes (IL10RB and IL18). The *P* values (based on parametric, unpaired, two-sample equal variance, two-tailed distribution) were calculated using Student’s *t *test for each gene in the control and treatment groups. Only genes with a *P*-value less than 0.05 and fold-change greater than 2.0 were considered significantly differentially expressed.

#### Western blotting.

Quantification was performed using ImageJ software using the integrated density of each band that was adjusted for background signal. The relative fold change shows values normalized to the control caudal disc RIPK3 signal.

## Supplementary Material

Appendix 01 (PDF)

Dataset S01 (XLSX)

## Data Availability

RNAseq data have been deposited in GEO (GEO: GSE173884) (56).
