# Supplementary material for: Nonpathological inflammation drives the development of an avian flight adaptation
Source: Proc Natl Acad Sci U S A. 2023 May 1;120(19):e2219757120. doi: 10.1073/pnas.2219757120 (PMC10175837; doi:10.1073/pnas.2219757120)
Supplement: Supplementary file 1 — Appendix 01 (PDF) [file pnas.2219757120.sapp.pdf]

PNAS

Supporting Information for
Nonpathological inflammation drives the development of an avian flight adaptation

Dana J. Rashid; Joseph R. Sheheen; Tori Huey; Kevin Surya; Jackson B. Sanders; John R. Horner; Jovanka Voyich and Susan C. Chapman

Corresponding author: Dana Rashid
Email: danarashid5@gmail.com

This PDF file includes:

Graphical Abstract
Fig. S1-S6
Tables S1-S7
Legend for Dataset S1
SI References

Other supporting materials for this manuscript include the following:

Dataset S1 (separate Excel file)

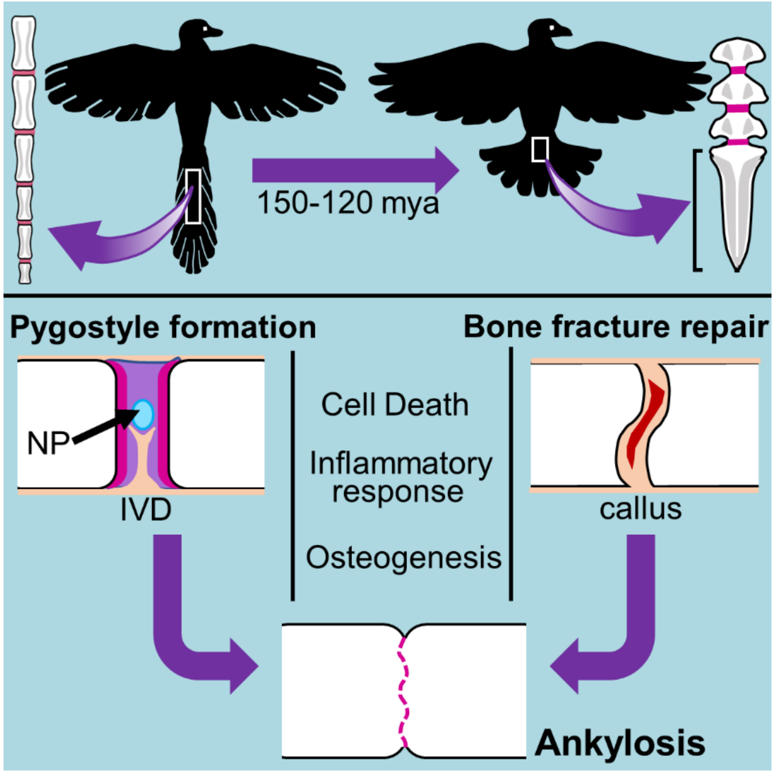

Graphical Abstract. Pygostyle formation, an adaptation first documented in Cretaceous avialans, persists in modern birds. Analysis of pygostyle formation in chickens indicates that this post-hatching process is driven by inflammation and closely resembles bone fracture repair.

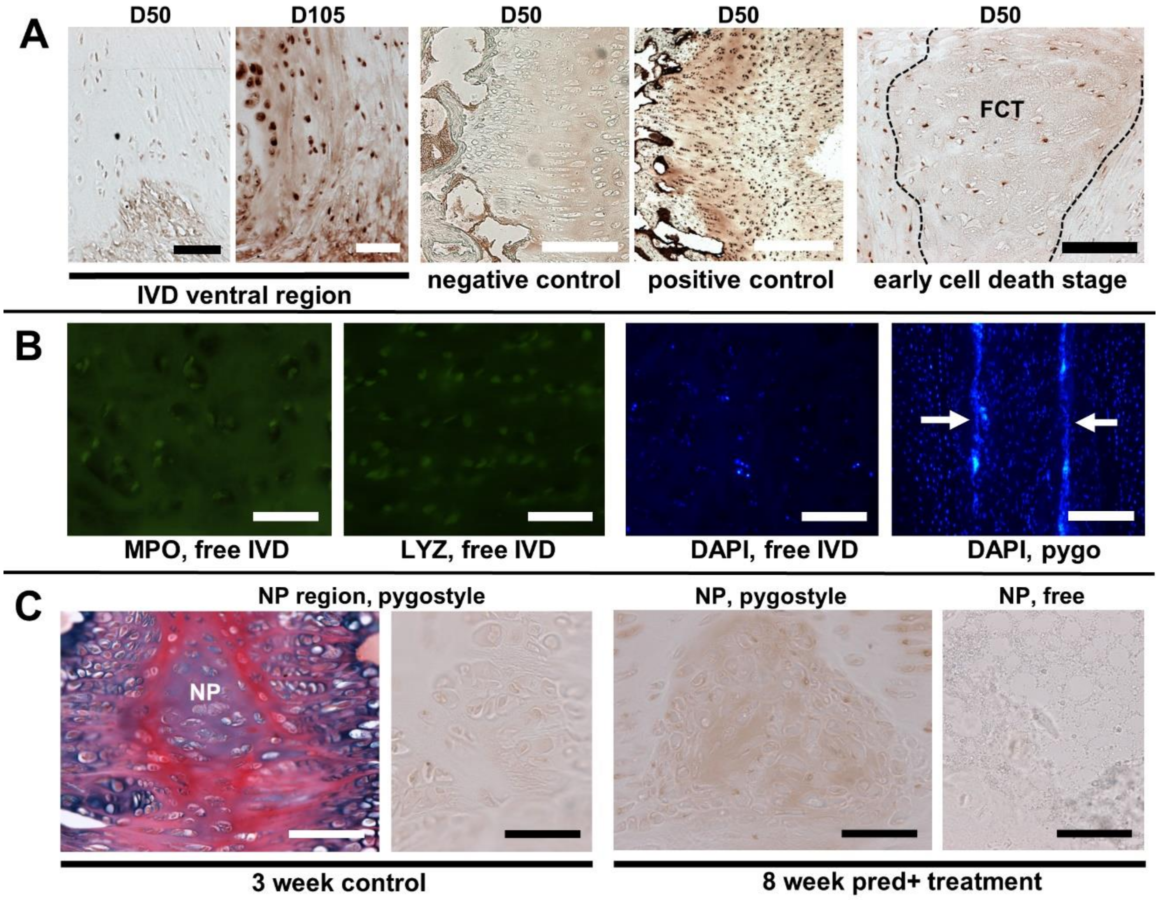

Fig. S1. Additional cell death, blood vessel, and nucleus pulposus data. A. TUNEL cell death progression data and controls. At D50 (left-hand panel), cell death was not observed in the ventral sides of pygostyle IVDs (scale bar 50 µm), but was detected in the fibrous connective tissue and immediately surrounding AF, suggesting that cell death initiates in the disc center. By D105, TUNEL-indicated cell death was evident throughout the discs, including in the ventral sides (scale bar 50 µm). In the two middle panels, the D50 negative (TdT enzyme omitted) and positive (DNAse-treated) TUNEL controls are shown (scale bars 200µm). In the far right panel, in the proximal disc of one D50 specimen, we observed scattered TUNEL-positive cells with no evidence of vascularization, suggesting cell death occurs before angiogenesis (scale bar 100µm). **B**. IHC controls and IVD blood vessel data. Neither MPO nor LYZ positive staining was observed in the centers of free, non-fusing IVDs (left-hand panels; scale bars 50 µm). This is consistent with the lack of blood vessels in free IVDs compared to fusing pygostyle IVDs, as shown with DAPI staining (right-hand panels; blood vessels noted by white arrows; scale bars 100µm). Two parallel-running blood vessels in pygostyle discs were commonly observed. **C**. Nucleus pulposus (NP) ontogeny and cell death for the prednisolone experiment (all scale bars, 50µm). Cellular NPs were observed at the onset of the prednisolone experiment in 3 week-old control tails (left-most panel, ABPRH staining). Negative TUNEL staining shows that at 3 weeks, the NP cells are still viable. Negative TUNEL staining of prednisolone-treated 8-week pygostyle and free tail NPs (right-hand panels) indicates that corticosteroid treatment preserves viable NP cells throughout the tail.

Table S1. RNAseq sample-wise sequencing and mapping statistics

	Free #1	Free #2	Free #3	Free #4	Pygo #1	Pygo #2	Pygo #3	Pygo #4
**Total sequenced reads passing RTA2 filter**	19192488	21193815	23466795	27411025	30908138	18665581	8770666	22480199
**Uniquely mapped reads STAR**	72.98%	71.45%	75.94%	77.21%	70.70%	72.61%	76.55%	73.63%
**Uniquely Assigned reads featureCount**	65.4%	62.3%	67.5	67.2%	59.6%	61.7%	64.8%	61.7%

Table S1 legend. Shown are the RNAseq filtering (by RTA2 filter), alignment (by STAR) and mapping (by SUBREADS featureCount) statistics for the four "Free" (unfused) and four "Pygo" (fusing pygostyle) IVD samples.

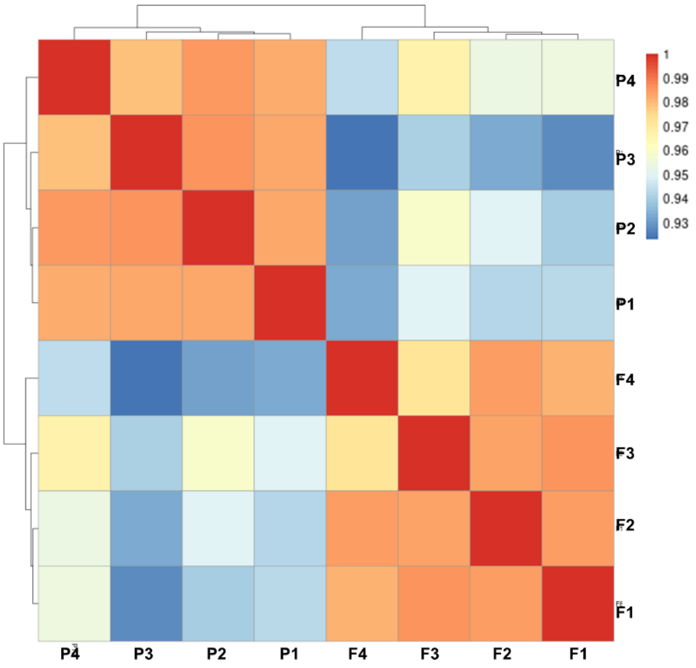

Figure S2. Hierarchical heatmap of all RNAseq samples. To ensure quality of the RNAseq biological replicates, a heatmap was generated using R. The heatmap shows relatedness clustering of the four pygostyle fusing IVD samples (P1-4), and the free non-fusing IVD samples (F1-F4). Deep red squares indicate identical samples (correlation of 1), darker blue indicate less correlation (correlation minimum 0.93). The base R function "cor()" was use to draw pairwise correlation values between variance stabilizing transformed (vst) data summarized for each sample with the DESeq2 assay() function, visualized by the pheatmap() function.

Table S2. KEGG analysis of RNAseq data

KEGG Pathway	Count	% matching genes	Genes	BH adjusted p-value
**gga04060:Cytokine-cytokine receptor interaction**	41	2.187834	CSF3R, AMHR2, CSF1, MPL, IL5RA, CXCR4, CSF2RB, TNFRSF11A, IL2RG, IL18RAP, CXCR1, CCL5, CCL4, IL21R, LOC100857191, IL12B, CCR7, CCL19, CCR6, CCR5, IL6R, CCR2, XCR1, IL15, IL1R2, TNFRSF18, TNFRSF19, LIFR, TNFRSF1B, PRLR, IL17RA, IL8L1, IL8L2, CXCL12, IL7, IL2RA, IL2RB, TNFRSF25, IL7R, BMPR1B, IL17B	4.89E-06
**gga04080:Neuroactive ligand-receptor interaction**	57	3.041622	GABRB3, VIPR1, CHRM4, MLNR, GRIK3, GRIK1, PTH1R, HTR4, ADRA1A, GRM3, PRLHRL, CYSLTR2, ADORA3, C3AR1, CTSG, UTS2R, GPR35, F2R, SSTR2, SSTR3, PRLR, TAAR1, CCKBR, ADORA2A, ADORA2B, AGTR1, PTGER4, GRIA1, PTGFR, CHRNA2, CHRNA4, C5AR1, PTAFR, LPAR3, PLG, ADRB2, MCHR2, P2RY8, GRIN2A, P2RY6, P2RY4, CNR1, HRH4, GPR156, S1PR4, GABRD, DRD3, GABBR2, P2RY10, HTR1F, GCGR, GZMA, GABRA3, GRIN2C, LPAR6, P2RX1, F2RL2	5.53E-05
**gga04145:Phagosome**	32	1.707577	COLEC12, ATP6V1A, NCF1, NCF2, ITGB3, NCF4, ITGB2, TCIRG1, MPO, THBS4, CTSS, C3, TUBA3E, TUBB1, DMB2, CD36, ATP6V1G3, ATP6V1E1, ATP6V1C2, ATP6AP1, BLB1, TAP2, TAP1, BF1, BF2, MARCO, BLA, ATP6V1B2, LOC420160, PLA2R1, TLR4, ATP6V0D2	3.39E-04
**gga04514:Cell adhesion molecules (CAMs)**	27	1.440768	CD86, NLGN1, SELPLG, NRXN1, CD80, ITGB2, CDH2, DMB2, ICOS, LRRC4C, ICOSLG, NTNG1, VCAM1, CADM1, ITGA4, BLB1, PDCD1LG2, BF1, BF2, CD2, SELP, CD4, PTPRC, CD8A, BLA, SIGLEC1, PDCD1	0.004429
**gga04620:Toll-like receptor signaling pathway**	21	1.120598	CD86, CD80, LY96, PIK3R1, MAPK12, PIK3R5, MAPK11, IL8L1, IL8L2, CTSK, CCL5, CCL4, SPP1, IRF7, IL12B, TLR7, IRF5, TLR5, IKBKE, TLR4, TLR3	0.018346
**gga04672:Intestinal immune network for IgA production**	11	0.586980	CD86, CXCL12, ITGA4, IL15, BLB1, BLA, CD80, CXCR4, DMB2, ICOS, ICOSLG	0.034245

Table S2. See legend below.

Table S3. GO analysis of RNAseq data

GO Biological Process	Count	% matching genes	Genes	BH adjusted p-value
**GO:0006955 immune response**	40	2.134471718	COLEC12, NRROS, OTUD7A, TNFRSFIIA, FYB, CTSS, OASL, CCLS, CCL4, ENPP2, BLNK, DMB2, CCR7, CCL1, CD36, CCR5, B2M, CCR2, SBSPON, CD74, BLB2, IL15, BLB1, TNFRSF18, TNFRSF1B, CD4, IL8L1, IL8L2, CXCL12, IGLL1, IL7, CCL17, IL2RA, BLA, XCL1, IRF8, TLR7, LCP2, CD244	3.05E-08
**GO:0006954 inflammatory response**	41	2.187833511	PTGER4P, TGFR, NRROS, C5AR1, PTAFR, TNFRSF11A, LIPA, PTGS1, C3, CCL5, CCL4, C3AR1, TLR21, CCR7, CCL1, CCL19, CCR5, CCL17, CCR2, LYN, GGT5, SYK, SLC11A1, TNFRSF1B, CD180,CYBB, CYBA, TNFRSF1B, SELP, ZAP70, IL8L1, IL8L2, CXCL12, IL2RA, TLR7, TLR5, BMPR1B, TLR4, TLR15, TLR3, IL17B	3.26E-07
**GO:0007169 transmembrane receptor protein tyrosine kinase signaling pathway**	24	1.280683031	LYN, RET, BLK, ITK, VCAM1, SYK, FLT3, TXK, INSRR, MATK, MST1R, ZAP70, HCK, DOK2, TEC, ERBB3, ERBB4, BLNK, BTK, STAP1, LCP2, ROR2, JAK3, PAG1	I.92E-05
**GO:0006935 chemotaxis**	15	0.800426894	LYN, PDGFRA, XCR1, PTAFR, CXCR4, LSP1, CXCR1, FES, ENPP2, C3AR1, RAC2, CCR6, CCR5, DOCK2, CCR2	4.44E-05
**GO:0050853 B cell receptor signaling Pathway**	14	0.747065101	LYN, BLK, VAV3, SYK, PRKCB, RFTN1, CD79B, ZAP70, TEC, KLHL6, INPP5D, PLCG2, PTPN6, NCKAP1L	4.46E-05
**GO:0045087 innate immune response**	31	1.654215582	BLK, ITK, TXK, HMGB2, NLRC5, JCHAIN, IFIH1, CATH1, PTK2B, TLR21, JAK3, B2M, LYN, SYK, MX1, MATK, CYBB, CYBA, CATH3, HCK, ZAP70, TEC, FES, BTK, TLR7, PTX3, TLR5, TLR4, S100A9, TLR3, SERINC5	I.95E-04
**GO:0050829 defense response to Gram-negative bacterium**	13	0.693703308	AVBD4, SLC11A1, HMGB2, RSFR, LY2, CATH3, CATH1, CD4, IL12B, AVBD7, TLR15, B2M, TLR4	I.97E-03
**GO:0038083 peptidyl-tyrosine Autophosphorylation**	14	0.747065101	LYN, BLK, ITK, SYK, TXK, MATK, ZAP70, HCK, TEC, ERBB4, FES, BTK, PTK2B, JAK3	0.0059826
**GO:0090023 positive regulation of neutrophil chemotaxis**	9	0.480256137	CD74, IL8L1, IL8L2, C3AR1, RAC2, CCR7, NCKAP1L, CCL19, THBS4	0.0059826
**GO:0060326 cell chemotaxis**	13	0.693703308	PDGFRA, DOCK4, PRKCD, C5AR1, HMGB2, CCL17, CCL5, CCL4, AGTR1, C3AR1, XCL1, PRKCQC, CL1	0.008967
**GO:0006952 defense response**	8	0.426894344	AVBD3, CD74, CD83, AVBD1, AVBD2, TAP1, LSP1, CATH3	0.0125819
**GO:0042742 defense response to Bacterium**	13	0.693703308	AVBD3, AVBD6, SYK, AVBD1, AVBD2, PRKCD, LY2, MPO, IL8L2, IRF8, TLR21, LYG2, TLR4	0.0333584
**GO:0030593 neutrophil chemotaxis**	11	0.586979723	VAV3, CATH1, CSF3R, IL8L2, SYK, ITGB2, CCL4, CCL1, NCKAP1L, CCL19, CCL17	0.0447868
**GO:0019370 leukotriene biosynthetic Process**	6	0.320170758	GGT5, SYK, ALOX5, ALOX5AP, LTA4H, LTC4SL	0.0626545
**GO:0070098 chemokine-mediated signaling pathway**	9	0.480256137	IL8L1, IL8L2, CXCL12, CCL5, CCL4, PTK2B, CCL1, CCL19, CCL17	7.29E-02

Tables S2 and S3 legend. RNAseq differential expression analysis of fusing vs non- fusing chicken tail IVDs. KEGG (Table S2) and GO (Table S3) pathway functional enrichment show extensive immune response during ankylosis. Pathways were ranked by count numbers whose BH adj p-value is less than 0.05, with a minimum Log_2FoldChange of 1 (2-fold change). Both enrichment tests were run on the upregulated genes in fusing chicken pygostyle IVDs compared to non-fusing IVD controls. The data was generated using the David Bioinformatics Functional Annotation tool.

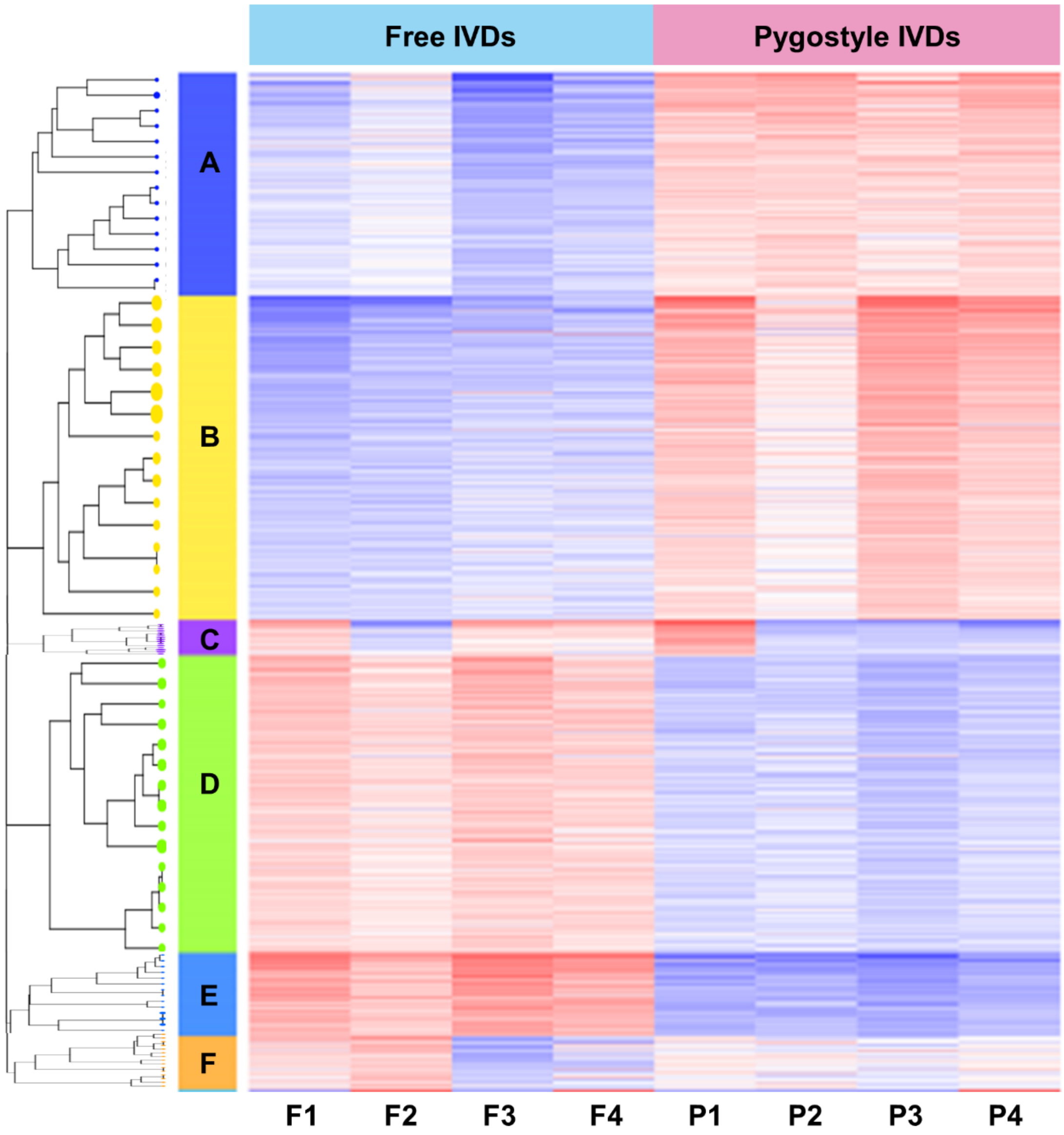

Figure S3. Non-hierarchical clustering heatmap for all RNAseq samples with GO annotation. K-means clustering of the top 1970 most variable genes (by standard deviation) in six groups was performed for all free non-fusing (F1-F4) and pygostyle fusing (P1-P4) IVD RNAseq samples. K-means clustering was performed using the R kmeans() function and normalized by gene mean center through iDEP.96 program (24,154 genes from the eight samples; 16,310 genes passed filter; VST; missing value=gene median). For the GO biological processes corresponding to the A-F clusters, see Table S4.

Table S4. **Corresponding GO Biological Processes for the Figure S3 heatmap**

Cluster	GO Biological Processes	# genes in Cluster
A	Osteoblast differentiation; Ossification; Connective tissue development; Cartilage development; Skeletal development; Biomineral tissue development; Response to BMP; Multicellular organism development; System development; Multicellular organismal process; Animal organ development; Anatomical structure morphogenesis; Animal organism morphogenesis; Cell communication; Signaling	427
B	Regulation of immune system process; Positive regulation of immune system process; Regulation of immune response; Immune system process; Activation of immune response; Leukocyte activation; Cell activation; Lymphocyte activation; Regulation of cell activation; lymphocyte proliferation; Mononuclear cell proliferation; Immune effector process; Defense response	643
C	Muscle tissue development; Striated muscle tissue development; Muscle cell development; Striated muscle cell development; Myofibril assembly; Cellular component assembly involved in morphogenesis; Actomyosin structure organization; System process; Muscle system process; Muscle contraction; Striated muscle contraction	72
D	Tissue development; Animal organ morphogenesis; Regulation of multicellular organismal process; Regulation of developmental process; Anatomical structure development; Developmental process; Multicellular organism development; System development; Multicellular organismal process; Anatomical structure morphogenesis; Regulation of cell component movement; Regulation of cell motility; Regulation of locomotion; Regulation of cell migration' Cell migration	547
E	Developmental process; Anatomical structure development; Multicellular organism development; System development; Multicellular organismal process; Anatomical structure morphogenesis; Biological adhesion; Cell adhesion; Transmembrane receptor protein serine/threonine kinase signaling pathway; Regulation of transmembrane receptor protein serine/threonine kinase signaling pathway; Extracellular structure organization; Extracellular matrix organization; External encapsulating structure organization; Collagen fibril organization; Metanephric proximal tubule development	164
F	Tube development; Tube morphogenesis; Vascular development; Blood vessel morphogenesis; Angiogenesis; Regulation of endothelial cell proliferation; Endothelial cell proliferation; Biological adhesion; Cell adhesion; Blood vessel diameter maintenance; Regulation of tube dimeter; Vascular process in circulatory system; Vasoconstriction	115

Table S4 Legend. For Figure S3 above, the GO biological processes corresponding to the A-F clusters and number of genes per cluster are noted.

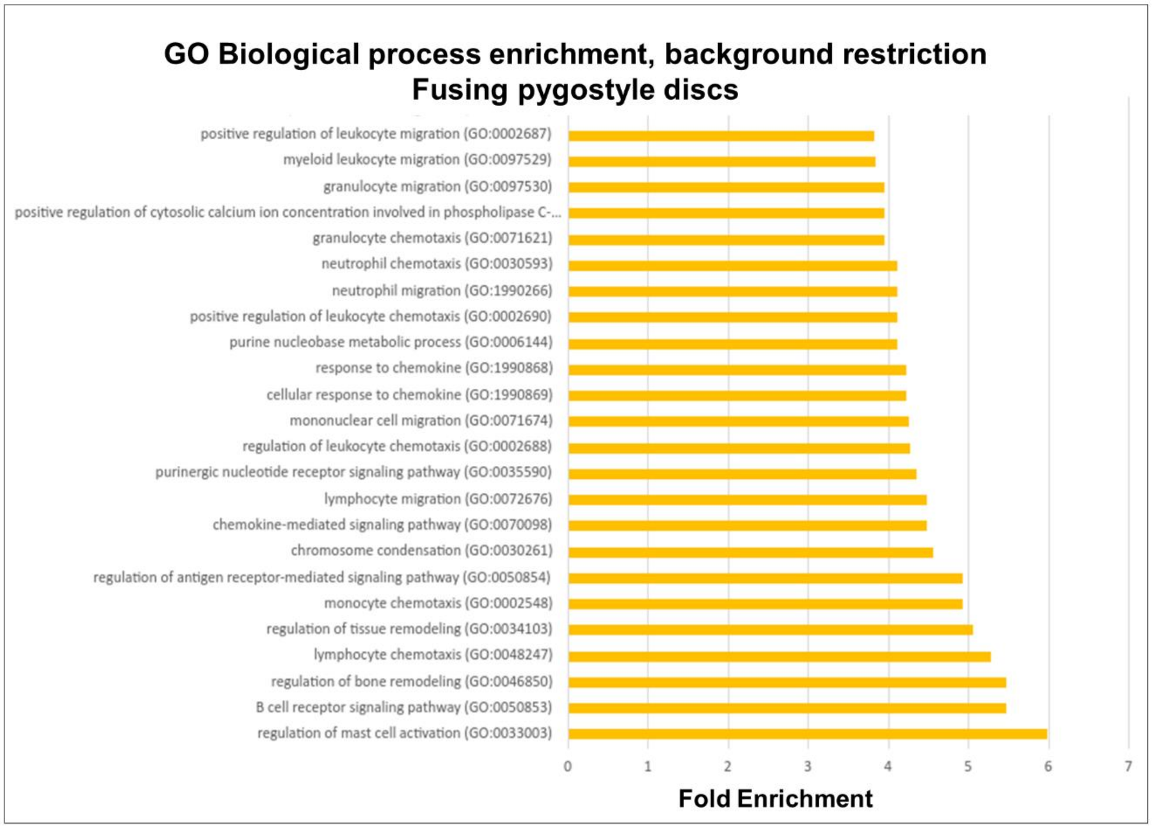

Figure S4. Top 25 GO biological processes of fusing pygostyle IVD transcripts with restricted background. The background/reference library consisted of the combined fusing and non-fusing IVD expressed genes (basemean ≥1) found in our RNAseq experiment (as opposed to the whole chicken genome). The reference background library was thereby reduced from the whole chicken genome (17,887 genes) to 13,332 genes. Fusing pygostyle and free IVD transcripts were analyzed against this restricted library, and the top 25 GO biological processes were determined using the PANTHER Overrepresentation Test. This analysis was conducted to normalize the enrichment analysis to IVD tissue only. The same overall trend was observed, substantiating significant immune response in fusing discs.

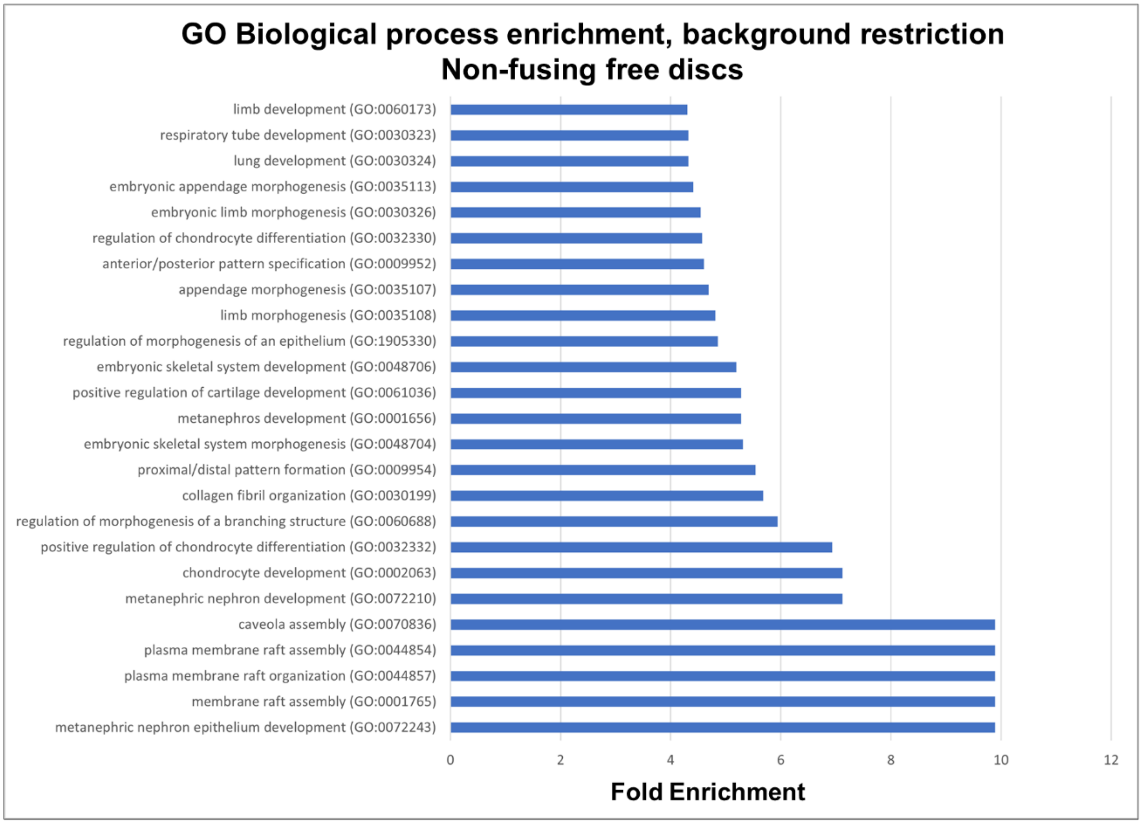

Figure S5. Top 25 GO biological processes of non-fusing free IVD transcripts with restricted background. As in Fig. S4 above, the background/reference library was the combined fusing pygostyle and non-fusing IVD genes (basemean ≥1). The non-fusing discs show enrichment of cartilage- and skeletal-specific processes, and lack an immune/inflammatory signature.

Table S5. Chicken immunoscreen RT-PCR array data

Gene	Gene Function	Fold Change	p-value
IL-5RA	IL-5 Receptor (Rc); eosinophil function	13.73	0.012
IL-8L1	Neutrophil, T cell, & macrophage chemotaxis	9.94	0.001
CXCR1	IL-8 Rc; neutrophil activation	7.42	0.001
SPP1	Osteopontin; bone matrix component; macrophage, T cell, & neutrophil activation; inducer of IL-17; wound healing	6.12	0.007
CCR5	Inflammatory CC chemokine Rc	4.83	0.005
CCR2	Inflammatory CC chemokine Rc	4.43	0.002
IL-2RG	IL-2 Rc subunit; neutrophil phagocytosis	4.25	0.001
CXCR4	CXCL12 Rc; bone fracture repair; fat signaling; cell migration	3.91	0.004
CXCL12	Bone fracture repair; T cell and monocyte chemotaxis	3.61	0.020
IL-17RA	IL-17A Rc; neutrophil maturation	3.46	0.003
TNFSF13B	B cell survival and immune response	3.38	0.017
IL-7R	Lymphocyte development	3.32	0.010
IL-17B	Stimulates release of IL-1beta and TNF alpha from monocytes	3.22	0.007
H6PD	Involved in macrophage phagocytic activity	3.09	0.002
CCL5	Chemoattractant for monocytes, T_H-1 cells, and eosinophils	2.86	0.039
LTB4R	Leukotriene Rc; fat-derived chemoattractant Rc	2.74	0.048
CCR7	Activates B andT lymphocytes; stimulates dendritic cell maturation	2.72	0.013
IL-18R1	IL-18 Rc; T_H-1 and Natural Killer cell immune response	2.36	0.004
CXCL14	Neutrophil attractant	-87.05	0.028
UBC	Ubiquitin; protein degradation	-13.77	0.000
AdipoQ	Fat metabolism; anti-inflammatory	- 7.18	0.009
VEGFA	Angiogenesis factor	- 3.87	0.010
CSF3	Induces granulocytes	- 2.91	0.048
TNFRSF11B	Inhibits osteoclasts	- 2.65	0.050
IL-1R1	IFNG production from T_H cells	- 2.61	0.014
CX3CL1	Adhesion of T cells and monocytes to endothelial cells	- 2.50	0.030
TNFSF4	Adhesion of T cells to endothelial cells	- 2.43	0.027
CSF2	Stimulates granulocytes & macrophages	- 2.28	0.028
IL-16	Blocks cell cycle progression in resting T cells	- 2.03	0.013

Table S5 legend. Up- and down-regulated genes (minimum fold change +/- 2; p values <0.05) from the screen of 8-week-old fusing chicken pygostyle IVD harvested RNA, including fold changes and p values. The fold changes are relative to non-fusing tail IVD controls. Evidence of both innate and adaptive immune response suggests immune involvement beyond the clearance of degenerating disc material. Gene functions were abbreviated from www.genecards.org.

Table S6. Comparison between pygostyle fusion RNAseq data with an Ankylosing Spondylitis (AS) mouse model microarray

Process	AS mouse array *(1)*	Pygostyle fusion
**Neutrophil Functioning**	Neutrophil cytosolic factor, Neutrophil granule protein, Neutrophil elastase, CSF3R, NCF4, CD177, CXCR2	Neutrophil cytosolic factor, Neutrophil granule protein, CSF3R, HCK, CXCR1, IL8L1, IL8L2, NCF2, NCF2, NCF4, GFI1, C3, ITGB2, LECT, S100A9, PI3, PRAM1, RAC2, SERPINB1
**Fat signaling**	Leukotriene B4 Rc, Phospholipase A2 (PLA2), Litaf (LPS-induced TN factor), PLPP3	Leukotriene C4 synthase, ALOXSAP, SLC01B1, PLA2R1, PLA2G10, PLAG10L, PLAG7, PLAG13, Litaf, MZB1, PLPPR4, ALOX15B, CYP4F11, TMPRSS3, PLEKHN1, PLEKHA2, GLIPR1L, CYSP1L, CPNE9, BIN2, TLR4, ABCG1, UGT8
**Complement pathways**	Complement C3, Cathepsin G	Upreg: Complement C3, C3aR1, C4, C4A, C5, C5aR1, C1Q/TNF6, CFH, Cathepsin G, perforin, ITGB2, MBL2, COLEC12, CFP, CD55, CR1L, CSMD2<br>Downreg: COLEC10, SERPING1, C1R, C15, VTN
**Toll-like Rc Signaling**	TLR2	Upreg: TLR3, TLR4, TLR5, TLR7, TLR15, TLR21, CD180, GFI1<br>Downreg: TLR2A
**ECM remodeling**	MMP3, MMP13, TIMP1	MMP13, ADAM19, ADAM33
**Cytokines/Chemokines and assoc. receptors and ligands**	CXCR2, CCL9, HCLS1 (LYN substrate), IL-1B	Upreg: CCL1, CCL4, CCL5, CCL17, CCL19, CCL26, CCR5, CXCR4, IL-1R2, IL-1RAPL2, IL-2RA, IL-2RB, IL-2RG, IL-4R, IL-5RA, IL-6R, IL-7, IL-7R, IL-8L1, IL-8L2, CXCR1, IL-12B, IL-12RB2, IL-15, IL-17B, Il-17RA, IL-17REL, Il-18R1, IL-18RAP, IL-21R, IL-22RA2, IL-31RA, ILD-R1, CXCR4, MZB1, CRCBL, LYN, TRPM2, CSF1R, CRLF2, FAM19A2<br>Downreg: IL-1R1, IL-1RAP, IL-1RL1, IL-11, IL-13RA1, IL-16, IL-17D, IL-18BP
**Cell death/apoptosis**	Caspase 8	CASP18, TP53I3, TNFRSF19, PDCD1, MZB1, CDK15, TMEM40L, MAP3K15, SERPINB10B, PTPRO, RASSF6, RIPK3, GPC3, TPD52L1, AT2, SOGA3, MLKL
**Osteogenesis**	Osteocalcin, Osteonectin, Col1a1, IBSP	Upreg: Osteomodulin, Osteocalcin, Osteoactivin, Col1a1, IBSP, CXCL12, PTH1R, ARSE, PHEX, IFITM5, MEPE, SIGLEC15, KZALD1, KL, PAX3, SPEF2, SVEP1, SATB2, LECT2<br>BMPs: BMP3, BMP5, BMPR1B, BMP8A, BMP2K, BMPER, FKBP1B, FLBP15
**Wnt Signaling**	Downreg: DKK1, SOST	Upreg: WNT9B, LGR5, WNT5B, SOST, DKK1, TNFRSF19<br>Downreg: WNT1, WNT5A, WNT9A

Table S6 legend. The 500 most up- and down-regulated genes of the pygostyle fusion RNAseq data were compared to a PGISp AS mouse model RNA microarray (1). Whole mouse spines were collected for the microarray, as opposed to fusing disc tissue for the pygostyle data, but numerous gene trends were evident. Listed genes are upregulated unless specified otherwise.

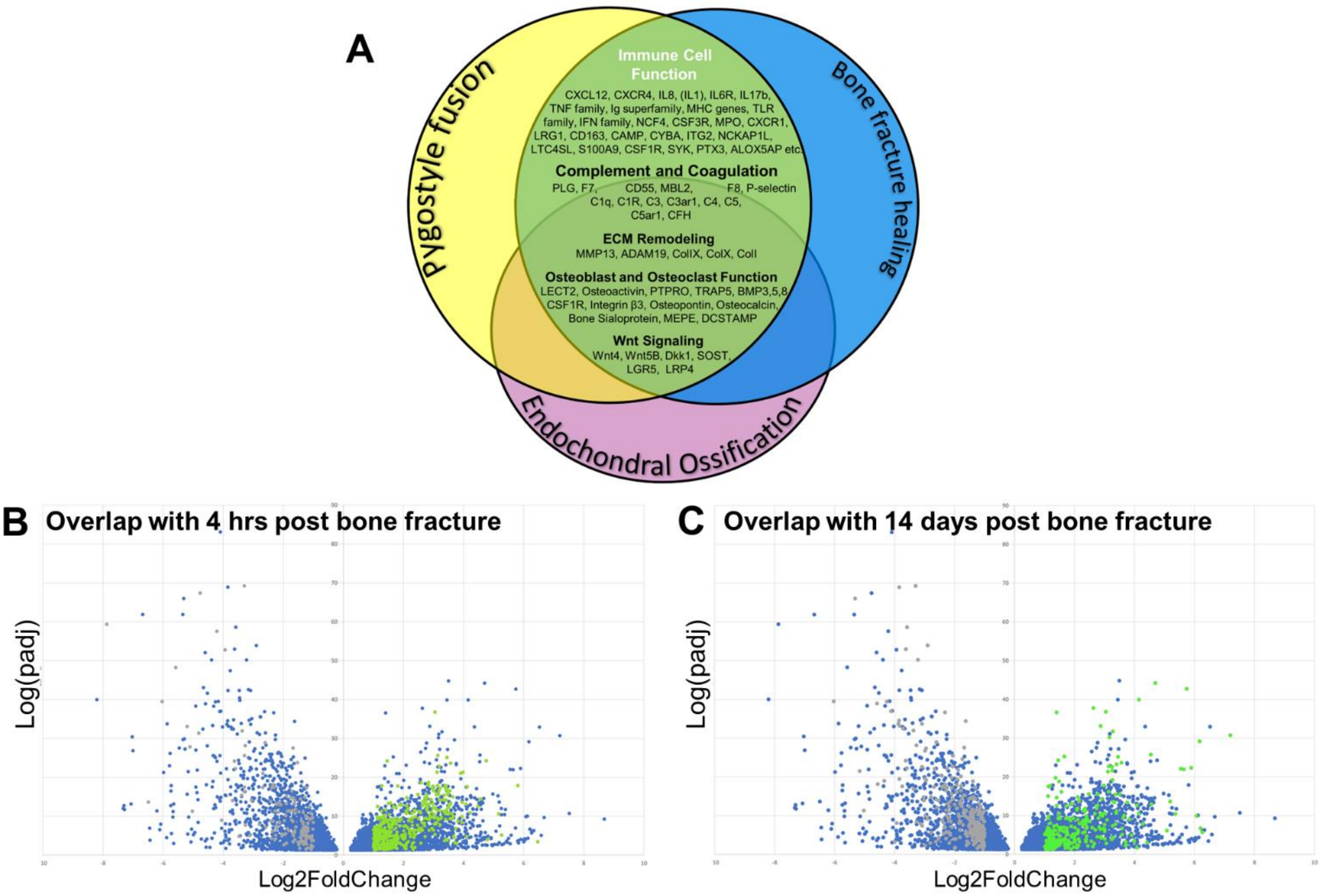

D

387 Shared Genes with 4 Hours Post Fracture
Fc receptor mediated stimulatory signaling pathway (GO:0002431)
Negative regulation of DNA duplex unwinding (GO:1905463)
Negative regulation of guanyl-nucleotide exchange factor activity (GO:1905098)
Platelet degranulation (GO:0002576)
Positive regulation of neutrophil extravasation (GO:2000391)
Regulation of neutrophil extravasation (GO:2000389)
Regulation of B cell receptor signaling pathway (GO:0050855)
Meiotic chromosome condensation (GO:0010032)
Double-strand break repair via break-induced replication (GO:0000727)
Mitotic chromosome condensation (GO:0007076)
Regulation of dendritic cell antigen processing and presentation (GO:0002604)
**155 Shared 4 hours Post Fracture Downregulated Genes - GO**
interleukin-33-mediated signaling pathway (GO:0038172)
7,8-dihydroneopterin 3'-triphosphate biosynthetic process (GO:0035998)
dihydrobiopterin metabolic process (GO:0051066)
leading edge cell differentiation (GO:0035026)
lactate transmembrane transport (GO:0035873)
lactate transport (GO:0015727)
metanephric nephron epithelium development (GO:0072243)
metanephric nephron tubule development (GO:0072234)
positive regulation of CD4-positive, alpha-beta T cell differentiation (GO:0043372)
tetrahydrofolate metabolic process (GO:0046653)
positive regulation of CD4-positive, alpha-beta T cell activation (GO:2000516)

E

296 Shared Genes with 14 Days Post Fracture - GO BP
positive regulation of mast cell chemotaxis (GO:0060754)
positive regulation of bone resorption (GO:0045780)
bone resorption (GO:0045453)
osteoclast differentiation (GO:0030316)
bone mineralization (GO:0030282)
actin filament bundle assembly (GO:0051017)
regulation of tissue remodeling (GO:0034103)
actin filament bundle organization (GO:0061572)
biomineralization (GO:0110148)
biomineral tissue development (GO:0031214)
tissue remodeling (GO:0048771)
**274 Shared 14 Days Post Fracture Downregulated Genes - GO**
positive regulation of epithelial to mesenchymal transition involved in endocardial cushion formation (GO:1905007)
regulation of epithelial to mesenchymal transition involved in endocardial cushion formation (GO:1905005)
positive regulation of cardiac epithelial to mesenchymal transition (GO:0062043)
regulation of cardiac epithelial to mesenchymal transition (GO:0062042)
endocardial cushion fusion (GO:0003274)
cell adhesion involved in heart morphogenesis (GO:0061343)
metanephric nephron tubule morphogenesis (GO:0072282)
bone trabecula morphogenesis (GO:0061430)
growth plate cartilage chondrocyte development (GO:0003431)
peptidyl-lysine hydroxylation (GO:0017185)
collagen biosynthetic process (GO:0032964)

Fig. S6. Pygostyle fusion exhibits endochondral ossification features and resembles bone fracture healing. A. Venn-type diagram comparison of genes upregulated in pygostyle fusion, bone fracture healing and non-trauma endochondral ossification. Upregulated pygostyle fusion genes were identified by our transcriptome profiling; upregulated bone fracture healing and endochondral ossification genes were curated from the literature (2-14). Inflammatory response and certain coagulation factors distinguish pygostyle fusion and bone fracture healing from non-injury endochondral ossification. Pertinent, but not all

shared genes are noted. **B, C.** Volcano plot comparisons of our pygostyle fusion transcriptome data versus a mouse bone fracture healing study (15) for the 4 days post fracture mouse transcriptome (**B**) and the 14 days post fracture mouse transcriptome (**C**). Green dots represent shared upregulated genes, grey dots represent shared downregulated genes, and blue dots represent unshared genes. The genes upregulated in our sequencing experiment that were shared with 4 hours and 14 days post fracture in the mouse bone fracture healing transcriptome were grouped into biological processes through the Gene Ontology Enrichment Analysis program (http://geneontology.org/). Each list of matching genes was uploaded into the tool separately and the top 11 most significantly enriched biological processes per timepoint were recorded in Fig. S6 D-E. All listed processes had false discovery rates below 0.05. Specific parameters were: Analysis Type: PANTHER Overrepresentation Test (Released 20210224); Annotation Version and Release Date: GO Ontology database DOI: 10.5281/zenodo.4495804 released 2021-02-01; Analyzed List:upload_1 (Gallus gallus), Reference List: Gallus gallus (all genes in database); Test Type: FISHER; Correction: FDR. The volcano plots, visualizing shared and unshared genes, were generated separately using Microsoft Excel. **D,E.** Tables showing the shared GO enrichment pathways from the comparisons performed in (B and C), respectively. The range of upregulated genes during pygostyle fusion, from enrichment of inflammation genes to enrichment of osteogenesis, is related to the combined maturational stages of the pygostyle IVDs that were collected from each pygostyle.

Table S7. Key Resources Table

REAGENT or RESOURCE	SOURCE	IDENTIFIER
**Biological samples**		
Gallus gallus 7-8 week Cornish Rock	Springdale Hutterite Colony, MT	N/A
Mus musculus, adult BALB/c	MSU Animal Resources Center	N/A
Dromaius novaehollandiae, Emu	Montana Emu Ranch, Kalispell MT	N/A
**Chemicals and Antibodies**		
Alcian Blue 8GX	Alfa Aesar	Cat# 33864-99-2
Cellulose Phosphate resin	Sigma Aldrich	Cat# C2258
DABCO	Sigma Aldrich	Cat# D27802
DPX mounting media	Electron Microscopy Sciences	Cat# 13510
Eosin Y	Electron Microscopy Sciences	Cat# 14851
Gill's Hematoxylin	Polysciences Inc.	Cat# 24243
Picrosirius red/Direct Red 80	Sigma Aldrich	Cat# 365548
Polyvinyl Alcohol (30-70K)	Sigma Aldrich	Cat# P8136
SYTOX Green	Molecular Probes	Cat# S7020
Wright Giemsa stain	Sigma Aldrich	Cat# WG16
NBT/BCIP Tablets (Roche)	Sigma Aldrich	Cat# 11697471001
Anti-Myeloperoxidase (MPO) PE-conjugated antibody	BD Biosciences	Cat# 333139
Anti-Lysozyme, hens egg, antibody AbD11397	Bio-Rad	Cat# HCA139A
Anti-RIPK3 antibody	MyBioSource	Cat# MBS3200470
Anti-GADPH antibody	Novus Biologicals	Cat# NB300-327
**Critical commercial assays**		
Agilent RNA 6000 Nano Kit	Agilent	Cat# 5067-1511
NEBNext® Poly(A) mRNA Magnetic Isolation Module	New England Biolabs	Cat# E7490
NEBNext® Ultra™ II RNA Library Prep Kit for Illumina®	New England Biolabs	Cat# E7775
RT2 PCR Profile Chicken chemokine PCR array	Qiagen	Cat# PAGG-011Z
In situ cell death POD kit (Roche)	Sigma Aldrich	Cat# 11684817910
Qubit RNA HS assay kit	ThermoFIsher Scientific	Cat# Q32852
NextSeq 500/550 Mid Output Kit v2.5 (300 Cycles)	Illumina	Cat# 20024905
**Deposited data**		
Raw and analyzed RNAseq data	this paper	GEO: GSE173884
**Experimental models: Organisms/strains**		
Gallus gallus/ White Leghorn	Charles River	SPF Premium Fertilized eggs
Gallus gallus/ Bovan Brown	Clemson University Poultry Farm	N/A
Gallus gallus/ Tetra Brown	Clemson University Poultry Farm	N/A
**Software and algorithms**		
GO (Gene Ontology)	*(16)*	http://geneontology.org
KEGG (Kyoto Encyclopedia of Genes and Genomes)	*(17)*	https://www.genome.jp/kegg/tool/map_pathway1.html
DAVID Functional Annotation Tool	*(18)*	https://david.ncifcrf.gov/summary.jsp
Spliced Transcripts Alignment to a Reference (STAR)	*(19)*	https://github.com/alexdobin/STAR/releases
SUBREAD featureCounts	*(20)*	http://subread.sourceforge.net/
DESeq2 R software package	*(21)*	https://bioconductor.org/packages/release/bioc/html/DESeq2.html
iDEP: Integrated Differential Expression and Pathway Analysis tool	*(22)*	http://ge-lab.org/idep/.
**Other**		
Mouse bone fracture healing transcriptomes	*(15)*	GEO:GSE152677
GeneCards, the Human Gene Database	*(23)*	www.genecards.org

Table S7 Legend. Pertinent resources utilized for this study are listed here.

Dataset S1 (separate file) Legend. The raw RNAseq data is presented in a multi-page Excel file. The pages include: 1. Original DE data; 2. padj (adjusted p value) filter; 3. >2 lfc (log fold change); 4. GO enrichment pygostyle (fusing IVDs); and 5. GO enrichment free (non-fusing IVDs).

SI References

1. Haynes KR *et al.* (2012) Excessive bone formation in a mouse model of ankylosing spondylitis is associated with decreases in Wnt pathway inhibitors. *Arthritis Research & Therapy* 14:R253-R253.
2. Hadjiargyrou M *et al.* (2002) Transcriptional profiling of bone regeneration. Insight into the molecular complexity of wound repair. *J Biol Chem* 277:30177-30182.
3. Gao F, Xu F, Wu D, Cheng J, Xia P (2017) Identification of novel genes associated with fracture healing in osteoporosis induced by Krm2 overexpression or Lrp5 deficiency. *Mol Med Rep* 15:3969-3976.
4. Skokowa J, Dale DC, Touw IP, Zeidler C, Welte K (2017) Severe congenital neutropenias. *Nature Reviews Disease Primers* 3:17032.
5. DeNichilo MO *et al.* (2016) Peroxidase Enzymes Regulate Collagen Biosynthesis and Matrix Mineralization by Cultured Human Osteoblasts. *Calcified Tissue International* 98:294-305.
6. Kovtun A *et al.* (2016) The crucial role of neutrophil granulocytes in bone fracture healing. *European Cells & Materials* 32:152-162.
7. Luo Wei LX *et al.* (2019) Alteration and significance of E-selectin and P-selectin in the tissues surrounding fracture in mouse models. *Chinese Journal of Tissue Engineering Research* 23:404-408.
8. Duncan EL, Brown MA (2010) Genetic Determinants of Bone Density and Fracture Risk—State of the Art and Future Directions. *The Journal of Clinical Endocrinology & Metabolism* 95:2576-2587.
9. Kokubu T, Haudenschild DR, Moseley TA, Rose L, Reddi AH (2008) Immunolocalization of IL-17A, IL-17B, and their receptors in chondrocytes during fracture healing. *The Journal of Histochemistry and Cytochemistry : official journal of the Histochemistry Society* 56:89-95.
10. Lin W *et al.* (2019) Lgr5-overexpressing mesenchymal stem cells augment fracture healing through regulation of Wnt/ERK signaling pathways and mitochondrial dynamics. *FASEB J* 33:8565-8577.
11. Chen Y *et al.* (2007) Beta-catenin signaling plays a disparate role in different phases of fracture repair: implications for therapy to improve bone healing. *PLoS Medicine* 4:e249.
12. Sarahrudi K, Thomas A, Albrecht C, Aharinejad S (2012) Strongly enhanced levels of sclerostin during human fracture healing. *Journal of Orthopaedic Research : official publication of the Orthopaedic Research Society* 30:1549-1555.
13. Hebb JH *et al.* (2018) Bone healing in an aged murine fracture model is characterized by sustained callus inflammation and decreased cell proliferation. *Journal of Orthopaedic Research : official publication of the Orthopaedic Research Society* 36:149-158.
14. Löffler J *et al.* (2019) Compromised bone healing in aged rats is associated with Impaired M2 macrophage function. *Frontiers in Immunology* 10:2443.
15. Bais M *et al.* (2009) Transcriptional Analysis of Fracture Healing and the Induction of Embryonic Stem Cell–Related Genes. *PLoS One* 4:e5393.
16. Ashburner M*, et al.* (2000) Gene Ontology: tool for the unification of biology. *Nature Genetics* 25(1):25-29.
17. Kanehisa M & Goto S (2000) KEGG: kyoto encyclopedia of genes and genomes. *Nucleic Acids Res* 28(1):27-30.
18. Huang da W, Sherman BT, & Lempicki RA (2009) Systematic and integrative analysis of large gene lists using DAVID bioinformatics resources. *Nature Protocols* 4(1):44-57.
19. Dobin A*, et al.* (2013) STAR: ultrafast universal RNA-seq aligner. *Bioinformatics (Oxford, England)* 29(1):15-21.
20. Liao Y, Smyth GK, & Shi W (2013) featureCounts: an efficient general purpose program for assigning sequence reads to genomic features. *Bioinformatics (Oxford, England)* 30(7):923-930.
21. Love MI, Huber W, & Anders S (2014) Moderated estimation of fold change and dispersion for RNA-seq data with DESeq2. *Genome Biology* 15(12):550.
22. Ge SX, Son EW, & Yao R (2018) iDEP: an integrated web application for differential expression and pathway analysis of RNA-Seq data. *BMC Bioinformatics* 19(1):534.
23. Stelzer G*, et al.* (2016) The GeneCards Suite: From Gene Data Mining to Disease Genome Sequence Analyses. *Current Protocols in Bioinformatics* 54(1):1.30.31-31.30.33.
